# Unearthing microbial diversity of *Taxus* rhizosphere via MiSeq high-throughput amplicon sequencing and isolate characterization

**DOI:** 10.1038/srep22006

**Published:** 2016-04-15

**Authors:** Da Cheng Hao, Si Meng Song, Jun Mu, Wen Li Hu, Pei Gen Xiao

**Affiliations:** 1 Biotechnology Institute, School of Environment and Chemical Engineering, Dalian Jiaotong University, Dalian 116028, China; 2 College of Marine Science and Technology, Zhejiang Ocean University, Zhoushan 316022, China; 3 Institute of Medicinal Plant Development, Chinese Academy of Medical Sciences, Beijing 100193, China

## Abstract

The species variability and potential environmental functions of *Taxus* rhizosphere microbial community were studied by comparative analyses of 15 16S rRNA and 15 ITS MiSeq sequencing libraries from *Taxus* rhizospheres in subtropical and temperate regions of China, as well as by isolating laccase-producing strains and polycyclic aromatic hydrocarbon (PAH)-degrading strains. Total reads could be assigned to 2,141 Operational Taxonomic Units (OTUs) belonging to 31 bacteria phyla and 2,904 OTUs of at least seven fungi phyla. The abundance of Planctomycetes, Actinobacteria, and Chloroflexi was higher in *T. cuspidata var. nana* and *T. *×* media* rhizospheres than in *T. mairei* rhizosphere (NF), while Acidobacteria, Proteobacteria, Nitrospirae, and unclassified bacteria were more abundant in the latter. Ascomycota and Zygomycota were predominant in NF, while two temperate *Taxus* rhizospheres had more unclassified fungi, Basidiomycota, and Chytridiomycota. The bacterial/fungal community richness and diversity were lower in NF than in other two. Three dye decolorizing fungal isolates were shown to be highly efficient in removing three classes of reactive dye, while two PAH-degrading fungi were able to degrade recalcitrant benzo[a]pyrene. The present studies extend the knowledge pedigree of the microbial diversity populating rhizospheres, and exemplify the method shift in research and development of resource plant rhizosphere.

The rhizosphere creates a multifaceted niche that could be adventured by myriad bacteria and fungi. The rhizosphere is the soil region influenced by root secretions, where complex microbial communities feed on nutrients from root exudates, mucilage, and sloughed-off root cells. This region is comprised of the rhizospheric soil tightly adhered to the roots, and the rhizoplane represented by the root surface^1^. Rhizosphere environments provide distinct and essential points of plant–microbial symbioses. The rhizosphere is regarded as a gold mine for the isolation of prospective biotransformation strains^2^,^3^ and potential biocontrol strains^4^, which have broad applications in agriculture, environment protection and remediation, and pharmaceutical industry, etc.

*Taxus* (yew) species such as *T. *×* media*, *T. cuspidata*, and *T. mairei* are important anticancer medicinal plants^5^. The regional inconsistency of *Taxus* rhizosphere bacterial community composition and diversity was revealed by comparative analysis of three 16S rRNA clone libraries from the *Taxus* rhizosphere in subtropical and temperate regions of China^2^. One hundred forty-six clones were obtained. Gammaproteobacteria, Betaproteobacteria, and actinobacteria were more abundant in *T. *×* media* rhizosphere of the temperate region than the subtropical *T. mairei* rhizosphere. Acidobacteria was more abundant in the *T. mairei* rhizosphere. The higher richness in the *Taxus* rhizosphere bacterial communities of the subtropical region was suggested, which now could be tested by the high-throughput sequencing approaches. By enrichment culture, a novel Actinobacteria strain DICP16 (*Leifsonia shinshuensis*) was isolated from the *T. *×* media* rhizosphere^2^,^6^, which was able to remove the xylosyl group from 7-xylosyl-10-deacetylbaccatin III and 7-xylosyl-10-deacetylpaclitaxel, thereby making the xylosyltaxanes available as sources of 10-deacetylbaccatin III and the anticancer drug paclitaxel. Nevertheless, the knowledge of the bacterial diversity of *Taxus* rhizospheres is still scarce, partially due to the low throughput of the traditional sequencing platform. Fungi play an active role in the interaction between plant genome and root microbiome^7^,^8^. The fungal diversity of *Taxus* rhizosphere is actually unknown, although there are some studies of fungal endophytes in *Taxus*^9^. Furthermore, the rhizosphere microflora of different *Taxus* species may have an important role in plant physiology and health; hence, an understanding of the microbial community structure of the rhizosphere is critical for the good agricultural practice (GAP) and biocontrol strains. Additionally, the knowledge of the microbial diversity of *Taxus* rhizospheres could facilitate the discovery of bacteria/fungi with strong biotransformation activity.

A high proportion of rhizosphere-inhabiting microbes cannot be cultured under the currently available artificial conditions. In recent years, culture-independent methods have evolved from the PCR-based technologies and Sanger sequencing to the next generation high-throughput sequencing, which has improved the detection, identification, and characterization of microbes directly from complex environmental samples such as rhizosphere soil^10^,^11^, circumventing the drawbacks of cultivation-based approaches. However, the culture-independent approach, which can provide myriad diversity information, has not been applied in most *Taxus* species and relevant microbial communities, let alone the high-throughput sequencing platform.

In the present study, the bacterial/fungal community structures of three *Taxus* rhizospheres in the subtropical and the temperate regions of China were explored using 16S rRNA/ITS (internal transcribed spacer) MiSeq high-throughput sequencing, followed by detailed bioinformatics and statistical analyses to explore the variability of microbial communities of *Taxus* rhizospheres in different species and environments. As far as we know, this is the first high-throughput amplicon sequencing study of bacterial/fungal community structure and diversity in the *Taxus* rhizosphere.

## Results

### Diversity estimation

*Taxus* rhizosphere samples, from five individual plants of each species, were collected from northeast (ZS, *T. cuspidata var. nana*, and MD, *T. *×* media*) and southeast (NF, *T. mairei*) China. The total number of 16S rRNA reads obtained from the 15 samples, after filtering chimeric sequences and mismatches, was 154,243, which were clustered into 2,141 operational taxonomic units (OTU) with at most 3% dissimilarity in nucleotide identity. The taxonomy and abundance of all OTUs are shown in Supplementary Table S1. The phylogenetic relationship of more than 400 bacterial genera that are identified in *Taxus* rhizospheres is shown in Supplementary Fig. S1. Rarefaction curves of most samples tend to be flat, suggesting that reasonable sequencing depth has been attained, although extra rare bacterial taxa are likely present in the sample. Shannon-Wiener curve (Figure available upon request) agrees with this claim. ACE and Chao1, the community richness index, of ZS and MD are comparable, and are higher than those of NF rhizosphere, implying that additional OTUs are likely present in NF, although coverage estimates were very high for all samples (Table 1). Both unrarefied and rarefied OTU richness (the number of OTUs in each sample before and after rarefying) are shown in Table 1. All bioinformatics results reported here were based on the rarefied OTUs. Both Shannon and Simpson diversity indices, which integrate evenness and species richness, suggest that taxonomic diversity of the bacterial community is higher in MD and ZS rhizospheres than in NF ones.

**Figure 1:**
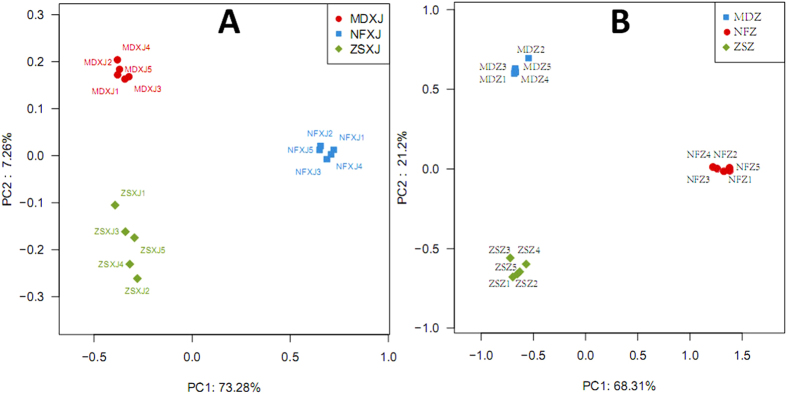
Comparison of microbial community in samples from different *Taxus* rhizospheres. Principal Coordinate Analysis (PCoA) was generated with OTUs (at 97% similarity) present in the different rhizosphere samples. (**A**) bacteria (XJ). (**B**) fungi (Z). PC, principal coordinate; MD, *T. *×* media*; ZS, *T. cuspidata var. nana*; NF, *T. mairei*.

The extent of variation of rhizospheric bacteria population in the different samples correlates with the natural habitat and *Taxus* species. The bacteria community composition of MD was similar to that of ZS, and these two are quite different from NF. In this study, XJ and Z represent bacteria and fungi respectively. In principal coordinate analysis (PCoA; Fig. 1A), the bacterial community composition of the sample ZSXJ1 was not only close to those of other four closely related ZS samples but also close to that of MD. MDXJ3 was more close to MDXJ1 and 2 than to MDXJ4 (Fig. 1A). PC1 alone explains 73.28% of variance (Fig. 1A), followed by PC2 (7.26%) and PC3 (2.4%).

The total number of ITS reads obtained from the 15 samples, after filtering quality control, was 333,972, which were clustered into 2,904 OTU with at most 3% nucleotide difference. The taxonomy and abundance of all OTUs are shown in Supplementary Table S2. The phylogenetic relationship of more than 100 fungal families that are identified in *Taxus* rhizospheres is shown in Supplementary Fig. S1. Rarefaction curves of most samples tend to be flat, suggesting that reasonable sequencing depth has been attained, which is supported by Shannon-Wiener curve (Figure available upon request). ACE and Chao1 of ZS rhizospheres are highest (Table 1), followed by MD, and these indices are lowest in NF samples, indicating that ZS has the best fungal community richness and additional OTUs are more likely present in NF. Both Shannon and Simpson diversity indices suggest that taxonomic diversity of the fungal community is higher in MD and ZS than in NF.

For the first time, this study reveals that *Taxus* rhizospheres are rich in fungi with variation across species and regions. The fungal community structure of MD was similar to that of ZS, both of which are distinct from NF. In PCoA (Fig. 1B), the fungal community composition of the sample MDZ1 was more close to that of MDZ4 than to MDZ2. NFZ1 was more close to NFZ2 and 5 than to NFZ4 (Fig. 1B). PC1 alone explains 68.31% of variance (Fig. 1B), followed by PC2 (21.2%) and PC3 (2.36%).

Adonis (permutational MANOVA) showed that there is significant difference between MD, ZS, and NF microbial communities (bacteria: R^2^ 0.801, *p* < 0.001; fungi: R^2^ 0.772, *p* < 0.001). Anosim, another non-parametric statistical test, suggested that the difference between MD, ZS, and NF microbial communities is substantially greater than the intra-group difference (bacteria: R 0.984, *p* < 0.001; fungi: R 0.999, *p* < 0.001).

### Taxonomic distribution of bacteria identified by MiSeq sequencing

**Figure 2:**
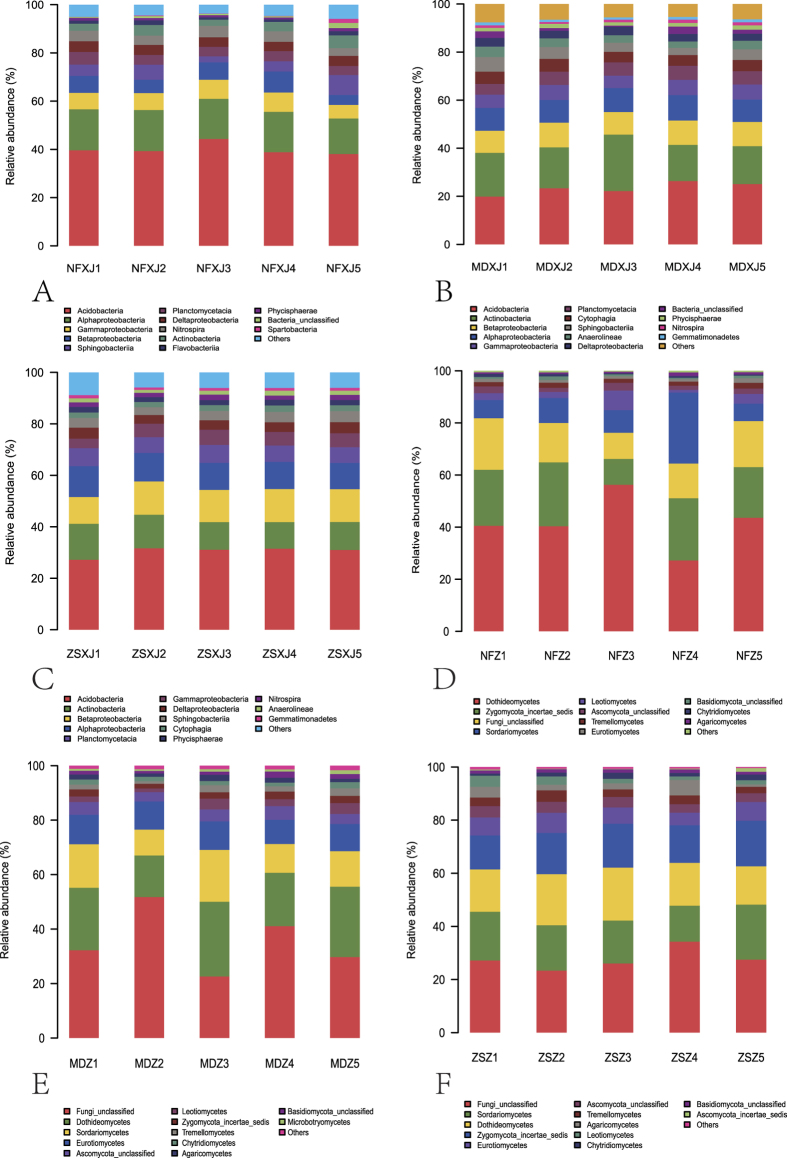
Relative abundance of different microbial classes in *Taxus* rhizosphere. (**A–C**) bacteria. The classes Sphingobacteria, Cytophagia, and Flavobacteria belong to Bacteroidetes; Phycisphaerae belongs to Planctomycetes; Anaerolineae belongs to Chloroflexi; Spartobacteria belongs to Verrucomicrobia. (**D–F**) fungi. Dothideomycetes, Eurotiomycetes, Leotiomycetes, and Sordariomycetes belong to Ascomycota; Tremellomycetes, Microbotryomycetes, and Agaricomycetes belong to Basidiomycota.

OTUs were further assigned to different taxa and their relative taxonomic abundance was estimated across the different rhizospheres. Seventy one bacteria classes belonging to at least 31 phyla were identified, including some unknown groups; the predominant phyla were Proteobacteria, Acidobacteria, Actinobacteria, Bacteroidetes, Planctomycetes, Nitrospirae, Chloroflexi, Gemmatimonadetes, and Verrucomicrobia. These major phyla were represented in all rhizospheres and accounted for a large number of reads. The Proteobacteria and Acidobacteria represented the highest number of reads in each rhizosphere, together making up at least 52% of the total bacteria population in each set of samples (Fig. 2A–C).

**Figure 3:**
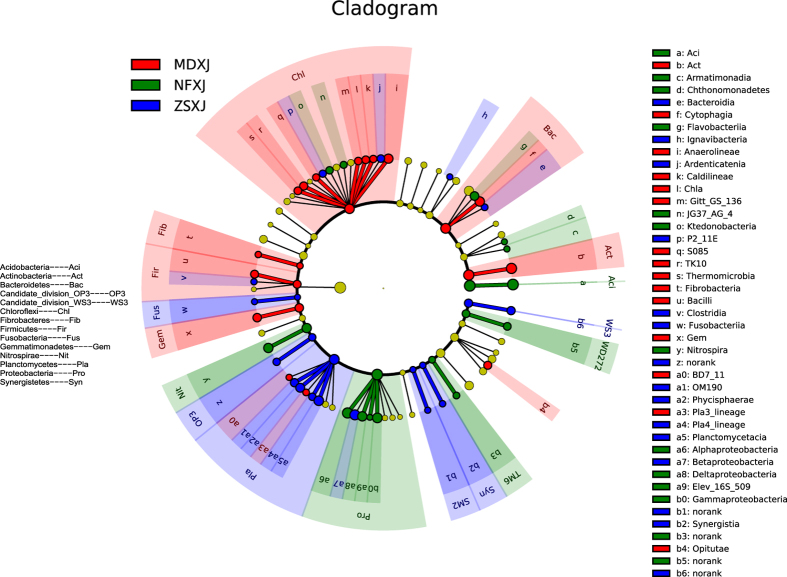
LEfSe results on *Taxus* rhizosphere microbiomes. The cladogram reports the taxonomic representation of statistically and biologically consistent differences between MD, ZS, and NF bacterial communities. Differences are represented in the color of the most abundant class (red indicating MD, green NF, blue ZS, yellow non-significant). Each circle’s diameter is proportional to the taxon’s abundance. This representation, employing the Ribosomal Database Project (RDP) taxonomy, simultaneously highlights specific phyla and classes.

LEfSe is a method for metagenomic biomarker discovery by class comparison, tests of biological consistency and effect size estimation^12^. The differential features were identified on the OTU level. The three rhizosphere groups were used as the class of subjects. LEfSe finds 14, 16, and 18 bacterial clades, of NF, ZS, and MD respectively, which consistently explain the statistically significant differences between three microbial communities (Fig. 3 and Supplementary Table S3). The most differentially abundant bacterial taxa in NF, ZS, and MD belong to Acidobacteria, Betaproteobacteria, and Actinobacteria, respectively (Figs 2A–C and 3). The overrepresented clades of NF also include Alphaproteobacteria, Gammaproteobacteria, and Deltaproteobacteria, which is different from those of ZS (Planctomycetacia, Phycisphaerae, and OM190 of Planctomycetes) and MD (Cytophagia, Anaerolineae, and Gemmatimonadetes), demonstrating the β-diversity of these communities. These results were approved by Metastats results at the genus level (not shown). The number of genera that exhibited significantly different abundance in NF, ZS, and MD is shown in Supplementary Fig. S2.

### Actinobacteria

Sequence reads of the Actinobacteria phylum could be classified into 14 orders and some unidentified and unclassified orders. Euzebyales, Glycomycetales, and Kineosporiales were represented by very few reads in MD and ZS, and were completely absent in NF. The Acidimicrobiales and Solirubrobacterales orders bore the most abundant reads in MD and ZS, followed by Propionibacteriales, Micrococcales (to which *Leifsonia shinshuensis* belongs), and Streptomycetales, while more reads belonging to Gaiellales were in NF (Supplementary Fig. S3). The Acidimicrobiales reads of MD and ZS rhizospheres mostly belong to the families Acidimicrobiaceae and Iamiaceae, as well as the uncultured and unclassified families (Supplementary Fig. S4 and Table S1). The Micrococcales reads of MD and ZS samples frequently belong to the families Microbacteriaceae, Intrasporangiaceae, and Micrococcaceae. Of note, the Microbacteriaceae reads of all samples belong to the genera *Agromyces*, *Lysinimonas*, *Microbacterium*, and the unclassified genera. No *Leifsonia* read was detected. Sixty seven Actinobacteria genera were identified, most of which had more reads in MD and ZS samples than in NF, with the exception of *Acidothermus* (Acidothermaceae, Frankiales). Some important Actinobacteria genera, e.g., *Arthrobacter*, *Frankia*, *Micromonospora*, *Mycobacterium*, *Nocardia*, and *Streptomyces*, were identified in the *Taxus* rhizospheres.

### Proteobacteria

Reads of the Proteobacteria could be assigned to four classes, i.e., Alpha-, Beta-, Delta-, and Gammaproteobacteria, along with some unidentified groups. These four classes were present in all rhizospheres. In NF, Alphaproteobacteria dominated over Gammaproteobacteria, followed by Beta- and Delta-proteobacteria (Fig. 2A). In MD and ZS, Betaproteobacteria had relatively high reads, followed by Alpha-, Gamma-, and Delta-proteobacteria (Fig. 2B,C). Alphaproteobacteria was represented by six orders and at least 44 families, including some uncultured and unclassified groups. Betaproteobacteria was represented by nine orders and 14 families, in addition to some unclassified groups. The majority of the Alphaproteobacteria reads belong to the orders Rhizobiales, Rhodospirillales, and Sphingomonadales (Supplementary Fig. S3), while most Betaproteobacteria reads were affiliated with the orders Burkholderiales and Nitrosomonadales, and the unclassified TRA3-20. Rhizobiales was mainly represented by the families Bradyrhizobiaceae, Hyphomicrobiaceae, and Xanthobacteraceae (Supplementary Fig. S4 and Table S1), etc. Burkholderiales was mainly represented by the families Burkholderiaceae and Comamonadaceae. The relative abundance of many families varied greatly among NF, MD, and ZS rhizospheres.

**Figure 4:**
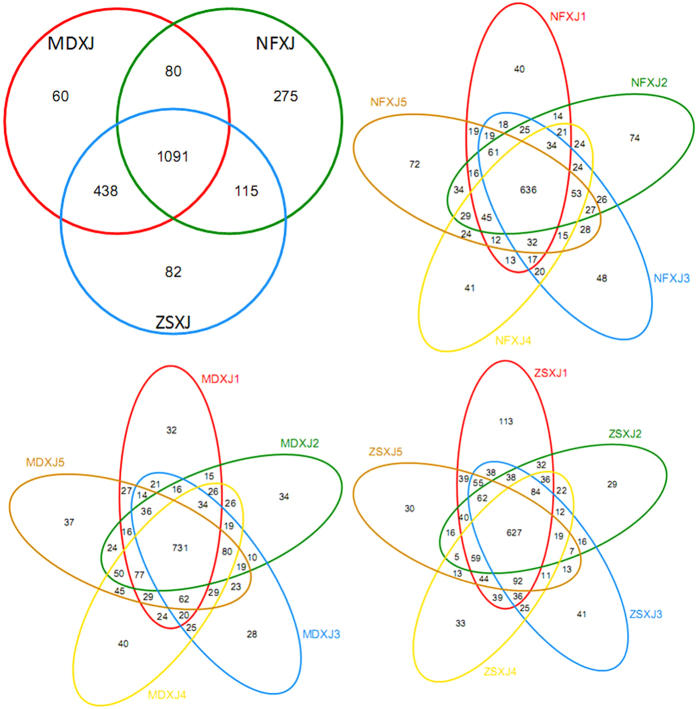
Venn diagrams showing the common and exclusive bacterial (XJ) OTUs of the *Taxus* rhizospheres. MD, *T. *×* media*; ZS, *T. cuspidata var. nana*; NF, *T. mairei*.

The Deltaproteobacteria of *Taxus* rhizospheres had eight orders and 18 families, and was mainly represented by the order Myxococcales (Supplementary Fig. S3), the unclassified GR-WP33-30, and Sh765B-TzT-29. The Myxococcales reads were dominated by the families Haliangiaceae and Sandaracinaceae (Supplementary Fig. S4 and Table S1), and some groups that could not be assigned to any known family. The relative abundance of Haliangiaceae in NF (0.479%), MD (0.378%), and ZS (0.383%) was comparable. The Gammaproteobacteria had at least 5.1% of the total reads in NF, MD, and ZS (Fig. 2), where it was comprised of 11 orders and at least 20 families. Xanthomonadales, the predominant order, was present in all samples. Together bacteria belonging to the Proteobacteria group account for 28.77%, 34.19%, and 32.16% of all reads in MD, NF, and ZS, respectively. The common and unique bacteria OTUs of *Taxus* rhizospheres are shown in Fig. 4.

### Taxonomic distribution of fungi identified by MiSeq sequencing

Twenty six classes belonging to at least seven phyla were identified, including some unknown groups; the predominant phyla were Ascomycota, Zygomycota, Basidiomycota, and Chytridiomycota. These phyla were represented in all rhizospheres. The Ascomycota had the highest number of reads in each rhizosphere, together making up at least 47% of the total fungi population in each set of samples (Fig. 2D–F). The common and unique fungi OTUs of *Taxus* rhizospheres are shown in Fig. S5. LEfSe finds 18, 26, and 25 fungal clades, of NF, ZS, and MD respectively, which consistently explain the statistically significant differences between three microbial communities (Supplementary Fig. S6 and Table S4). The most differentially abundant fungal taxa in NF, ZS, and MD belong to Dothideomycetes (Ascomycota), Pleosporales (Dothideomycetes, Ascomycota), and Unclassified, respectively (Fig. 2D–F and S6). The overrepresented clades of NF also include Mortierellales (Zygomycota), Xylariales (Sordariomycetes, Ascomycota), and Venturiales (Dothideomycetes, Ascomycota), which is different from those of ZS (Hypocreales, Basidiomycota, and Sordariales of Ascomycota) and MD (Chaetothyriales, Dothideales, and Eurotiales of Ascomycota), indicating the β-diversity of these communities. These results were approved by Metastats results at the genus level (not shown). The number of genera that exhibited significantly different abundance in NF, ZS, and MD is shown in Supplementary Fig. S2.

### Ascomycota

Sequence reads of Ascomycota could be classified into eight classes in addition to some unidentified classes. Across the different rhizospheres, Pezizomycetes, Saccharomycetes, and Orbiliomycetes were generally represented by very few reads and Lecanoromycetes were completely absent in NF. The Dothideomycetes class bore the most abundant reads in NF and the second most abundant reads in MD (Fig. 2D,E). The mean relative abundance of Sordariomycetes was not significantly higher in ZS (17.18%; Fig. 2F) than in MD (13.63%) and NF (11.84%) (ANOVA, *p* 0.353), so was that of Leotiomycetes (ZS 2.40%, MD 2.77%, NF 3.44%; *p* 0.639). In contrast, the mean relative abundance of Eurotiomycetes was considerably higher in MD (10.08%) and ZS (6.46%) than in NF (1.17%) (*p* < 0.001).

The dominating Dothideomycetes orders were Pleosporales, Dothideales, Venturiales, and Capnodiales (Supplementary Fig. S3). The abundant Hypocreales, Xylariales, and Sordariales belong to Sordariomycetes. Chaetothyriales and Eurotiales were predominant Eurotiomycetes orders, while Helotiales dominated Leotiomycetes. There were nine, nine, and eight dominating orders in MD, ZS, and NF, respectively. The prevalent orders of different rhizospheres are distinct. For instance, Pleosporales was shared by MD, ZS, and NF, while Capnodiales and Dothideales were shared by MD and ZS. ZS (9.92%) had significantly higher relative abundance of Hypocreales than MD (6.62%) and NF (5.78%) (*p* 0.016). The mean relative abundance of Eurotiales was considerably higher in MD (3.47%) and ZS (1.87%) than in NF (0.59%) (*p* < 0.001).

**Figure 5:**
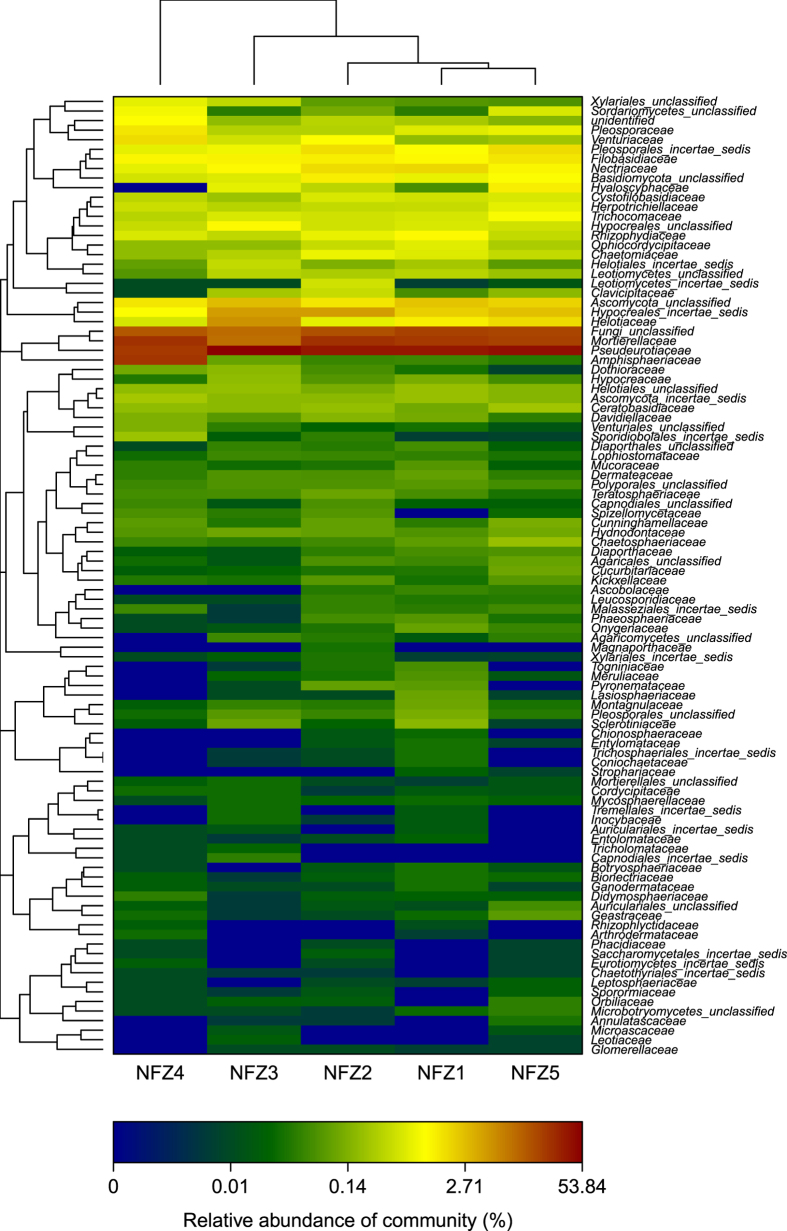
Heat maps showing fungal family frequency distribution in NF. The top 100 abundant families are shown. The different color intensities represent the relative fungal abundance in each rhizosphere. The clustering along y axis is based on abundance of family reads present in each rhizosphere; the clustering along x axis is based on the similarity of the inter-sample abundance.

There were 12, 12, and 13 dominating families in MD, ZS, and NF, respectively. The relative abundance of the same family varied greatly in different rhizospheres. For example, the mean relative abundance of Trichocomaceae (Eurotiales) was significantly higher in MD (3.47%) and ZS (1.87%) than in NF (0.59%) (*p* < 0.001; Figs 5, S7 and S8), so was that of Nectriaceae (Hypocreales; MD 2.63%, ZS 2.55%, NF 1.37%; *p* 0.038). Glomerellaceae (Sordariomycetes; Figs 5 and S8), to which a newly isolated dye-decolorizing *Glomerella* strain (patent submitted) belongs, was one of the 169 fungal families identified in the MiSeq sequencing. Among 2,904 fungal OTUs, OTUs 1113, 1281, 1879, and 2681 belong to the family Glomerellaceae (Supplementary Table S2). OTUs 952, 1428, 1655, 1934, 2019, and 2473 belong to the genus *Myrothecium* (family *Incertae sedis*), from which a dye-decolorizing *M. verrucaria* strain was isolated (see below). OTU 1926 belongs to the genus *Talaromyces* (family Trichocomaceae), from which a dye-decolorizing *T. stollii* strain and a PAH (polycyclic aromatic hydrocarbon)-degrading *T. verruculosus* strain were isolated (see below). OTUs 386, 610, 1631, 1904, 1941, and 2187 belong to the genus *Fusarium* (family Nectriaceae), from which a PAH-degrading *F. oxysporum* strain was isolated (see below).

### Basidiomycota

Seven classes were identified. Ustilaginomycetes, Exobasidiomycetes, Atractiellomycetes, and Cystobasidiomycetes were of low abundance. Tremellomycetes and Agaricomycetes predominated in ZS, MD, and NF (Fig. 2D–F), while Microbotryomycetes predominated in MD. At the level of order, the mean relative abundance of Cystofilobasidiales was similar in ZS (0.77%), MD (0.99%), and NF (0.47%) (*p* 0.114; Supplementary Fig. S3). Leucosporidiales (Microbotryomycetes) was abundant only in MD. Polyporales, to which a newly isolated PAH-degrading *Abortiporus biennis* strain of our lab belongs, was identified in ZS (relative abundance 0.013%), MD (0.006%), and NF (0.11%). Among 2,904 fungal OTUs, OTU 2172 was found to be *A. biennis* (Supplementary Table S2).

### Functional potential of *Taxus* rhizosphere

**Figure 6:**
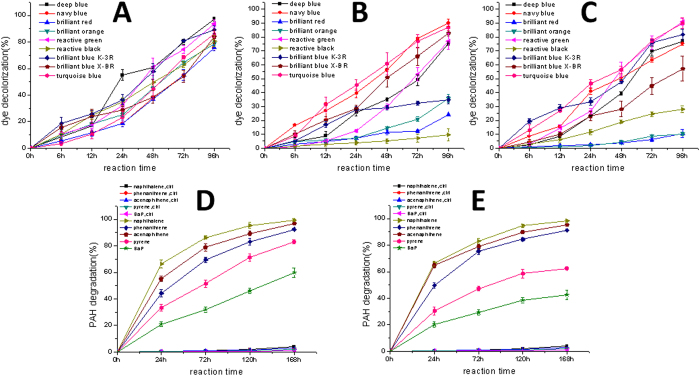
Functional diversity of *Taxus* rhizosphere fungi. Decolorization of nine reactive dyes by three laccase-producing fungi isolated from *Taxus* rhizospheres: (**A**) *Myrothecium verrucaria*; (**B**) *Glomerella*; (**C**) *Talaromyces stollii*. PAH degradation by two fungi strains isolated from *T. mairei* rhizosphere. (**D**) *Talaromyces verruculosus*; (**E**) *Abortiporus biennis*. Ctrl, controls without fungal cells. Error bars are standard deviations (n = 3).

Three dye decolorizing fungal strains were isolated from the enrichment culture, i.e., a *Myrothecium verrucaria* (Sordariomycetes, Ascomycota) strain DJTU-sh7 from MD, a *Glomerella* (Sordariomycetes) strain from ZS, and a *Talaromyces stollii* (Eurotiomycetes, Ascomycota) strain from NF. Fungal OTUs that are very similar to these strains have been detected in the high throughput amplicon sequencing (see above). A *M. verrucaria* strain NF-05 produces the laccase at a high yield and displayed the dye decolorizing ability^13^. The whole cell biotransformation approach was thus used to compare the dye decolorizing capacities of three strains (Fig. 6A–C). The strain DJTU-sh7 was able to decolorize all nine reactive dyes within 96 h (Fig. 6A), with the best performance in reactive deep blue (97.34% ± 1.38%), followed by reactive green (93.60% ± 2.99%). The *Glomerella* strain could efficiently decolorize five reactive dyes (Fig. 6B), but the decolorization of the other four dyes was poor, especially the reactive black (9.43% ± 4.35%). The decolorization abilities of the *Talaromyces* strain against different dyes varied greatly (Fig. 6C), with the best performance in reactive green (91.01% ± 2.55%) and reactive turquoise blue (89.88% ± 3.88%), and the worst performance in reactive brilliant orange (10.23% ± 1.16%) and reactive brilliant red (10.54% ± 3.12%).

Three PAH-degrading fungi were isolated from NF, i.e., a *Talaromyces verruculosus* (Eurotiomycetes) strain DJTU-SJ5, an *Abortiporus biennis* (Agaricomycetes, Basidiomycota) strain, and a *Fusarium oxysporum* (Sordariomycetes) strain. Fungal OTUs that are identical or very similar to these strains have been detected in the high throughput amplicon sequencing (see above). The *Fusarium* strain could only tolerate 50 mg/L pyrene in the initial screening and was not studied further. No pyrene-degrading bacteria were found in the screening. In the whole cell biotransformation, the strain DJTU-SJ5 could efficiently degrade all five PAHs within 168 h (Fig. 6D), with the best performance in the two-ring PAH naphthalene (99.51% ± 1.06%), followed by the three-ring PAHs (phenanthrene 92.38% ± 0.73%, acenaphthene 96.88% ± 0.15%) and the four-ring PAH pyrene (83.2% ± 1.46%). Importantly, DJTU-SJ5 could efficiently degrade 60% five-ring PAH benzopyrene, which is notorious for its recalcitrance. The *Abortiporus* strain could efficiently degrade two-ring and three-ring PAHs (Fig. 6E), but its ability in transforming four-ring and five-ring PAHs was not as good as DJTU-SJ5.

## Discussion

The genus *Taxus* is composed of the well-known source plants of anticancer paclitaxel (Taxol) and other useful taxanes^14^,^15^. *Taxus* associated microbes not only are involved in the taxane biosynthesis^16^,^17^,^18^,^19^ and the biotransformation of taxane compounds^2^, but also have other functional potentials. For instance, the phosphate-solubilizing bacteria were isolated from *T. mairei* rhizosphere^20^, which could be used to improve the growth of the seedlings. *Streptomyces* sp. En-1, endophytic to *T. chinensis*, produces phytohormone indole-3-acetic acid^21^. The antimicrobial agent-producing bacterium was isolated from *T. baccata* rhizosphere^22^, which was antagonistic against clinically significant microbes. Fungal endophytes have multiple functions. For example, a Taxol-producing endophyte induces transcription of genes encoding a redundant fungicide pathway in its host *Taxus* plant^23^, thus preventing colonization of its fungal competitors at minimal metabolic cost. In *T. baccata*, 15 fungal isolates out of the 77 isolated strains displayed antimicrobial activity^24^. *Perenniporia tephropora*, an endophytic fungus of *T. mairei*, had strong anti-*Pyricularia oryzae* activity and produced the cytotoxic metabolites^25^. The endophytic fungi of *T. sumatrana* exhibited anti α-glucosidase and anti-oxidative activities^26^. *Taxus* rhizospheres contain both bacteria and fungi with potential utilities, but they have not been fully explored. The fast-growing high-throughput DNA sequencing could help gain a comprehensive understanding of the microbial community structure in *Taxus* rhizospheres, which, complemented by the culture-dependent method^2^,^27^, would help developing strategy of sustainable utilization of rhizosphere microbial resources.

This study presents a culture independent analysis of the microbes associated with *Taxus* rhizospheres of different localities and multiple species. As far as we know, this is the first high-throughput sequencing study of the bacteria/fungi present in *Taxus* rhizospheres. *Taxus* rhizosphere appears to have a relatively higher diversity of bacteria when compared to the rhizospheres of *Pinus tabuliformis*^28^, *Robinia pseudoacacia*, and *Salix babylonica*^29^, and cultivable rhizosphere bacteria of *Rauwolfia* spp.^30^. The fungal diversity is also high in our *Taxus* rhizospheres, but direct comparisons of OTU richness between different studies might not be appropriate due to differences in sequencing and bioinformatics approaches. For instance, several hundred to a few thousand ITS OTUs and 5-30 AMF (arbuscular mycorrhizal fungi) OTUs are expected in a sample^29^. AMF are just a small group of fungi, and the Shannon indices of the rhizospheric AMF of 20 medicinal plant species, which was based on the spore quantification and identification, are quite low^31^. Thus obviously the entire fungal community is much more diverse. However, it should be noted that only 187 fungal OTUs were revealed as the *Taxus chinensis* root-associated endophytes^32^. The Shannon indices of the *Taxus* rhizosphere are comparable to those of the *Pinus tabuliformis* rhizosphere^28^, but the very high Simpson indices of the latter suggest the low community diversity. On the other hand, a high throughput analysis of the fungal population of *Fritillaria thunbergii* rhizospheres revealed higher Ace, Chao, and Shannon values^33^, implying the influences of plant species and edaphic factors. Although the Shannon and Chao indices of the *Taxus* rhizosphere bacteria of this study are lower than those of the *T. chinensis* root-associated bacteria^32^, the number of bacterial OTUs of the two studies is 2,141 and 913 respectively. These results suggest the abundant microbial diversity of our rhizosphere samples and the utility of MiSeq amplicon sequencing in uncovering such diversity.

The diversity of bacteria inhabiting *Taxus* rhizosphere varied across different regions and species. The bacteria community composition was similar across different samples of the same locality and species, but greatly differed from the different ones (Figs 1 and 2). ZS is more similar to MD than to NF, which might be influenced by their habitat and the host species. Previous studies of *Taxus* rhizospheres and endophytes have shown that bacteria diversity profiles associated with different species of different habitats were different^2^,^32^. Acidobacteria and Alphaproteobacteria were predominant in both wild and cultivated *T. mairei* rhizospheres of Lishui, China^2^, implying that the tree age and physiology influence bacterial diversity of *Taxus* rhizosphere. Previously only 146 clones were screened for three 16S rRNA clone libraries of two *Taxus* species^2^, and the rhizosphere community richness and diversity cannot be delineated accurately based on such small sampling. Our study presents Acidobacteria as the most abundant phylum in NF, and Proteobacteria as the most plentiful phylum in ZS and MD. This switch of the most abundant bacteria group may result from the change in habitat and species. In *T. chinensis* of Jiangxi, China, more root-associated OTUs, mainly endosphere bacteria, were from Proteobacteria (63.24%) than from Acidobacteria (14.35%), Bacteroidetes (7.83%), and Actinobacteria (7.18%)^32^, which is distinct from the rhizosphere community structures of the temperate *Taxus* and the subtropical *T. mairei*. At the class level, the Proteobacteria OTUs of *T. chinensis* were largely from Alpha- (25.67%), Gamma- (20.75%) and Betaproteobacteria (13.38%). Although this sorting order is similar to that of NF, the respective percentage in NF is much smaller than that in *T. chinensis*. Analogously, the rhizosphere community structures of MD and ZS, which were from the Dalian study sites and are closely connected, are by no means identical. For instance, both have unique OTUs except the shared OTUs (Fig. 4); Planctomycetacia is more abundant in ZS than in MD, while Cytophagia (Bacteroidetes) in reverse; Oxalobacteraceae and Comamonadaceae are dominating Burkholderiales (Betaproteobacteria) families in MD and ZS respectively (Supplementary Fig. S4 and Table S1). The plant species growing closely connected could host distinct root-associated bacterial communities^34^. *T. *×* media* (MD) is a hybrid between *T. cuspidata* and *T. baccata*, while *T. cuspidata var. nana* (ZS) is a variety of *T. cuspidata*. Their different identities could structure the root-associated communities. In addition, our results support that the rhizosphere and endosphere, two compartments of the root-associated microbiomes, display dissimilar but overlapping microbial communities^1^,^35^.

Some rhizobacteria have plant growth promoting potential, e.g., they are functional in the solubilization of inorganic phosphate^20^ and production of indole acetic acid^21^. Some rhizobacteria produce xylosidase^6^, laccase, and anti-fungal chemicals^36^. The applicability of the *Taxus* rhizosphere bacteria could be underscored as a reliable component in sustainable agriculture. Further mining of versatile functional potentials in engineering and industry is warranted to boost the rational utilization of the *Taxus* rhizosphere resource.

To date, some studies of *Taxus* endophytes have been reported^18^,^19^,^37^, with the aims of finding paclitaxel-producing fungi. There are some studies of the community structure of *Taxus* associated fungi^9^,^17^,^32^. However, the fungal profile of *Taxus* rhizospheres has not been outlined, and whether some of the endosphere fungi can be found in rhizosphere is not known. Lack of knowledge of *Taxus* rhizosphere fungi is disadvantageous for the sustainable utilization of *Taxus* resources. The metagenomic analysis of *T. chinensis* root-associated microbes detected 14 bacterial classes and 19 fungal classes^32^, while our amplicon sequencing identified 71 bacterial classes and 26 fungal classes. ITS, with much better resolving power, is a more commonly used fungal marker than 18S rRNA, and thus much more fungal OTUs (2,904) were identified via ITS amplicon sequencing rather than via 18S amplicon sequencing (187). Ascomycota is by far the most dominant phylum in NF, MD, and ZS of this study, which was also number one phylum in *T. globosa*^9^, *T. mairei*^17^, and *T. *×* media* endophytes^18^. Contrarily, Basidiomycota was the most dominant phylum in *T. chinensis* root-associated fungi^32^, and the dominant genera are distinct, which might be due to the tissue, species, and habitat specificities. Sequences belonging to 3,380 fungi genera were identified in the metagenomic analysis of *T. chinensis* root^32^, among which 36 genera are reported to have Taxol-producing species. Intriguingly, 21 of these 36 genera, e.g., *Chaetomium*, *Alternaria*, and *Trichoderma*, are present in our *Taxus* rhizospheres (Supplementary Table S5). Two additional genera *Hypocrea* and *Gibberella*, which were taxane-producing endophytes of *T. mairei*^38^, are also identified in our rhizosphere samples. These encouraging results enable further endeavors in utilizing the underexplored rhizosphere resource to find new Taxol-producing fungi.

Some Ascomycota strains produce laccase with high yield, and thus have potential applications in environmental protection, e.g., decoloring the wastewater^39^, as well as biomass conversion^40^. Some Ascomycota and Basidiomycota strains are active degraders of PAHs^41^,^42^. A few such strains have been isolated from our *Taxus* rhizospheres (patent submitted), suggesting that various functional potentials except taxane production could be mined from rhizosphere resource. The global view gained from MiSeq sequencing is helpful in fostering the expanded utilization of *Taxus* rhizospheres.

Many abundant fungi species associated with *Taxus* rhizospheres were either uncultured or unclassified. Accordingly, the distribution of fungal OTUs is more discrete compared to that of bacterial OTUs, i.e., more unique fungal OTUs are identified from MD, ZS, and NF, respectively, validating the power of the MiSeq high-throughput sequencing in disclosing novel and/or rare taxa. Similar to the bacterial profile, ZS and MD of the adjacent study sites share more fungal species, including some rare ones that cannot be found by the current culture-dependent techniques, e.g., those of the genera *Dokmaia*, *Myriangium*, and *Simplicillium* (Supplementary Table S6). It is likely that many rare fungi are specialized rhizosphere fungi of the temperate *Taxus*, which might have particular role in the rhizosphere ecology and physiology. Future work looking at the transcriptomic and proteomic patterns of these fungi among *Taxus* rhizospheres and modes of function would be the next step in understanding the importance of these particular fungi and possibly their susceptibility to handling.

## Conclusion

Bacterial and fungal communities in the plant rhizospheres are central to the host health, survival and growth, and are also a treasure bowl rich in natural resources for potential applications in agriculture and industry. Richness estimates and diversity indices of three sets of libraries revealed major differences, indicating a higher richness in MD and ZS rhizospheres than in NF, and considerable variability in the bacterial/fungal community structure and composition within each habitat and species. Since the samples were not only from different species, but also from different environments, it is impossible to conclude whether the clear differences in bacterial and fungal community composition are due to *Taxus* species or environment. Given the intricate interrelationship between plant and its root-associated microbial assemblage, the rhizosphere microbiome could be regarded as an extension of the plant genome. This work has enabled a deeper understanding of the bacterial/fungal colonizers in different *Taxus* rhizospheres. Some important inhabitants that were previously not known to be in the gymnosperm rhizosphere have been highlighted, such as those overlooked by our previous Sanger sequencing, and more importantly, the putative Taxol-producing fungi and other microbes with many promising uses. The *Taxus* rhizospheres harbor a large diversity of bacteria representing 31 phyla and fungi of at least seven phyla. Shifts of the microbial groups along the axis of habitat and host are unambiguous, possibly due to the selection pressure during the coevolution of the host plant and rhizosphere microbes. This study provides new clues on rhizosphere microbes that could be used to investigate the linkage between bacterial and fungal rhizosphere communities, between microbes and host plant, and between rhizosphere microbes and environmental factors. Information contributed by this study could be exploited in developing valuable programs and reagents for agriculture and industry. Understanding the specific functions and the ecological patterns of common and rare species will be a fruitful area for future research.

## Methods

### Sample collection and DNA extraction

*Taxus* rhizosphere samples were collected from two regions of China, in May 2014. The age of *Taxus* plants was between 10–20 years. Roots and large clumps of non-rooted soil were removed. Rhizosphere samples were collected from five individual plants of each species within the field of 100 m^2^. The five individuals were around 5–10 meters far from each other. All plants used in this study were field-cultivated. The samples were stored at −80 °C before nucleic acid analyses: MD, *T. *×* media* rhizosphere of Dalian Institute of Chemical Physics (38.92°N, 121.62°E), Northeastern China; ZS, *T. cuspidata var. nana* rhizosphere of Dongbei University of Finance and Economics (38.92°N, 121.61°E), Dalian, Northeastern China; NF, *T. mairei* rhizosphere of Nanjing Botanic Garden (32.04°N, 118.78°E), Jiangsu, Southeastern China. The site at Dalian has a temperate climate, and is at an altitude of 50 m. The forest at Nanjing is in a subtropical region, and at an elevation of 200 m. The E.Z.N.A.^®^ Soil DNA Kit (Omega Biotek) was used to extract genomic DNA from rhizosphere soil samples. DNA was checked by agarose gel electrophoresis and its purity was examined by NanoDrop spectrophotometer.

### PCR, amplicon quatification, MiSeq library construction and sequencing

The variable region V4-5 of the 16S rRNA gene was selected for the construction of the bacterial community library for MiSeq sequencing. The broadly conserved primers, 515F, 5′-GTGCCAGCMGCCGCGG-3′, and 907R, 5′- CCGTCAATTCMTTTRAGTTT-3′, were used for PCR to amplify the sequencing region of the 16S rRNA gene. PCR primers for amplifying fungal ITS1-2 are: ITS1-1737F, 5′-GGAAGTAAAAGTCGTAACAAGG-3′; ITS2-2043R, 5′-GCTGCGTTCTTCATCGATGC-3′.

PCRs was carried out in 20 μL reactions in triplicate, with each reaction tube containing 0.2 mM of each primer, 10 ng of template DNA, 0.25 mM dNTPs, 1 × PCR reaction buffer, 2 U of FastPfu DNA Polymerase (ShineGene, Shanghai, China). The following PCR condition was used for 16S rRNA: 95 °C for 2 min, 95 °C 30 sec, 55 °C 30 sec and 72 °C 45 sec for 30 cycles, and a final extension of 72 °C for 10 min. The same PCR conditions were used for ITS, except that the second stage had 35 cycles. PCR products were subsequently subjected to electrophoresis on a 2% agarose gel, stained with ethidium bromide, and the targeted fragment size (16S rRNA 392 bp, ITS 306 bp) purified with an AxyPrepDNA gel extraction kit (Axygen, China).

Prior to MiSeq sequencing, the concentration of the purified PCR product was checked and quality controlled using a QuantiFluor^TM^-ST fluorometer (Promega) and an Agilent 2100 bioanalyzer (Agilent, USA). After quantification, equimolar ratio from each mixture were pooled and subjected to clonal amplification for generating amplicon libraries. TruSeq DNA LT Sample Prep Kit (Illumina) was used to construct paired-end (PE) sequencing libraries. MiSeq Reagent Kit v2 (Illumina) was used to perform amplicon sequencing on a MiSeq Desktop Sequencer.

### Isolation of laccase-producing fungi and dye decolorization via respective fungal isolates

- Enrichment and screening of laccase-producing microbes. *Taxus* rhizosphere soil samples (10 g) were dried for 6 h at 50 °C and then put in 100 mL sterile water with glass beads, which was cultured over night at 30 °C and 130 r/min. Ten mL of the above suspension were put in 100 mL enrichment culture medium (sucrose 30 g, CuSO_4_•5H_2_O 0.5 g, NaNO_3_ 2 g, MgSO_4_•7H_2_O 0.5 g, K_2_HPO_4_ 1.0 g, FeSO_4_ 0.01 g, KCl 0.5 g, 1 L) and shaked for 5 d at 30  °C and 130 r/min. One mL of enrichment culture was mixed with 9 mL sterile water to obtain the suspension of 10^−1^ dilution. The suspensions of 10^−2^–10^−7^ dilution were sequentially prepared. One hundred μl of diluted suspension (10^−3^–10^−7^, triplicate) were plated on the screening medium (PDA medium, agar 15 g, 0.04% guaiacol) and incubated for 7 d at 28 °C. The single colonies of brown red color were picked out and streak-cultured for 7 d. The pure microbial strains were inoculated and preserved on the slope of PDA culture medium (20% potato, 2% glucose, penicillin 50 μg/ml, streptomycin 100 μg/ml, KH_2_PO_4_ 3 g, agar 2%, MgSO_4_-7H_2_O 2 g, VB1 trace, 1 L) at 4 °C.
- Re-screening of fungi. The fungal strains from the above screening were grown in the PDA medium for 7 d at 28 °C, which was followed by the liquid culture at 30 °C and 130 r/min for 7 d in 100 mL laccase-producing medium (glucose 20 g, ammonium tartrate 10 g, KH_2_PO_4_ 2 g, MgSO_4_•7H_2_O 0.5 g, anhydrous CaCl_2_ 0.075 g, CuSO_4_•5H_2_O 0.01 g, 1 L). The fermentation mixture was centrifuged at 12,000 r/min for 5 min to collect the supernatant as the crude enzyme. The laccase activity of the crude enzyme was determined and the fungal strains with higher laccase activity were chosen for the following study.
- Identification of the fungal strains. The Universal Genomic DNA Extraction Kit Ver 5.0 (Takara, Dalian, China) was used to extract the genomic DNA. PCR primers used to amplify ITS (internal transcribed spacer) sequences are: ITS1-1737F, GGAAGTAAAAGTCGTAACAAGG, and ITS2-2043R, GCTGCGTTCTTCATCGATGC. The PCR reaction mixture (25 μL) contains 10 × buffer 2.5 μL, dNTP (2.5 mM each) 1 μL, 1 μL of each primer (10 μM), Ex Taq DNA polymerase (5 u/μL) 0.25 μL, genomic DNA 1 μL, and ddH_2_O 18.25 μL. PCR reaction conditions: 95 °C, 5 min; 94 °C 30 s, 56 °C 1 min, and 72 °C 3 min of 35 cycles; 72 °C, 5 min. The purified PCR products were subjected to the DNA sequencing (Takara, Dalian, China). The BLAST of NCBI GenBank was used to search for the similar sequences. The ITS sequence alignment was performed by Clustal ω and the phylogenetic tree was constructed by MEGA 6 software (http://megasoftware.net/mega.php).
- Measurements of the laccase activity. The reaction mixture of 10 mL consists of 0.04 mmol guaiacol in 1 mL 95% ethanol, 1 mL crude enzyme, and 8 mL sodium succinate buffer, which react for 30 min at 30 °C and are subjected to absorbance determination at 465 nm. The inactivated crude enzyme (boil for 5 min) was used as the negative control. One unit of the laccase activity was defined as the enzyme amount that is used to catalyze the oxidation of 1 nmol guaiacol within 1 min. In laccase activity (U/mL) = 10^6^ × reaction volume × ΔA/(volume of crude enzyme × absorbance coefficient ε × Δt), ΔA is the change of absorbance and Δt is the reaction time.
- Decolorization of reactive dyes. Six azo dyes (reactive deep blue M-2GE, reactive navy blue B-GD, reactive brilliant red KE-7B, reactive brilliant orange K-GN, reactive green 19, and reactive black 5) were purchased from Sigma Company. Two anthraquinone dyes (reactive brilliant blue K-3R and X-BR) and reactive turquoise blue KN-G were from Shanghai Jiaying Chemical Engineering Co. Ltd. The degradation of nine structurally different dyes by the fungi suspension culture or the crude laccase was determined by full spectrum scan among 400–700 nm between 0–96 h. The dye decolorization was calculated at various time points. The reaction mixture for the standard assay contained respective dye (20 mg/L) in 10 mM citric acid-Na_2_HPO_4_ buffer at pH 4.5 and 1 mL fungal suspension in a total volume of 10 ml. The dye decolorization (%) = [(Ai−At)/Ai] × 100, where Ai: initial absorbance of the dye, At: absorbance of the dye along the time. All experiments were performed in triplicate. The maximal absorbance wavelength of these dyes is 612 nm (reactive deep blue), 596 nm (reactive navy blue), 565 nm (reactive brilliant red), 479 nm (reactive brilliant orange), 632 nm (reactive green), 597 nm (reactive black), 623 nm (reactive brilliant blue K-3R), 596 nm (X-BR) and 601 nm (reactive turquoise blue), respectively.

### Isolation of PAH-degrading fungi and PAH degradation via respective fungal isolates

- Screening of PAH-degrading microbes. Two grams of *Taxus* rhizosphere soil were mixed with 18 mL sterile water and were incubated at 28 °C and 150 r/min for 24 h. Two mL of the above mixture were put into inorganic salt culture medium (pH 7.2 phosphate buffer 6 mL, MgSO_4_•H_2_O 0.0735 g, CaCl_2_ 0.001 g, FeCl_3_ 0.002 g, ZnSO_4_•H_2_O 0.002 g, MnSO_4_•H_2_O 0.5 mg, 1 L) that contained pyrene of 0.05 g/L as the sole carbon source, and were shaked at 28 °C for 5 d. The concentration of pyrene was then increased to 0.1 g/L and 0.2 g/L for further domestication. 0.1 mL of the domesticated enrichment culture was plated on the screening medium (K_2_HPO_4_ 2 g, KH_2_PO_4_ 0.6 g, pyrene 0.3 g, NaCl 0.5 g, NH_4_NO_3_ 1.5 g, MgSO_4_•7H_2_O 0.05 g, MnSO_4_•H_2_O 0.015 g, FeSO_4_•7H_2_O 0.01 g, CaCl_2_ 0.01 g, agar 20 g, 1 L) and were incubated at 28 °C for 5 d. The single colonies of PAH-degrading microbes were picked out and streak-cultured for 7 d.
- The identification of the fungal strains was similar as described for dye decolorizing fungi.
- Measurements of PAH degradation. PAH-degrading strains were grown in the seed medium (beef extract 3.0 g, peptone 10.0 g, NaCl 4.0 g, 1 L) at 25 °C and 130 r/min for 48 h, then the culture mixture was centrifuged at 6000 r/min for 5 min, and the pellet was rinsed thrice with the phosphate buffer. Five mL fungal suspension was prepared for the following degradation experiments. The reaction mixture of 50 mL contained the fungal suspension of 0.1mL, PAH (pyrene, final concentration 100 mg/L; phenanthrene, 200 mg/L; naphthalene, 200 mg/L; acenaphthene, 200 mg/L; benzopyrene, 100 mg/L), 300 mg/L Tween 80, and inorganic salt medium. Each experiment was repeated thrice at 28 °C and 150 r/min for 7 d. The supernatant was obtained by centrifuging the reaction mixture at 6000 r/min for 25 min. C18 solid phase microextraction column (Agilent) was used to extract degradation products from the filtered supernatant, with dichloromethane as the elution solvent and the elution rate 2 mL/min. The residual PAH substrate was quantified with the UV absorbance at 252 nm (pyrene), 289 nm (phenanthrene), 283 nm (naphthalene), 309 nm (acenaphthene), or 295 nm (benzopyrene). The standard curves were used to calculate the PAH degradation (%).

### Bioinformatics and statistical analysis

The raw sequencing reads of this study were submitted to the National Center for Biotechnology Information Sequence Read Archive under accession no. SRP051525. PE reads were overlapped to form contiguous reads. Softwares Trimmomatic and FLASH were used in quality control and filtering. The sequences were then clustered into OTUs by Usearch 7.1^43^ (http://qiime.org/) based on 97% pairwise identity using QIIME’s^44^ open reference OTU picking strategy. Taxonomic classification of the representative sequence for each prokaryotic OTU was performed using the Ribosomal Database Project classifier (Release 11.1 http://rdp.cme.msu.edu/) against the Greengenes 16S rRNA database (Release 13.5 http://greengenes.secondgenome.com/) and Silva (Release 115 http://www.arb-silva.de). The fungal ITS database in Unite (Release 5.0 http://unite.ut.ee/index.php) was used for fungal OTUs. All OTUs identified as belonging to chloroplast and mitochondria were removed from the data set. Chimeric OTUs were identified using uchime (version 4.2.40 http://drive5.com/usearch/manual/uchime_algo.html) and removed from the OTU table. The phylogenetic trees were generated from the alignment file by FastTree (version 2.1.3 http://www.microbesonline.org/fasttree/).

ZSXJ2 had the lowest number of reads, i.e., 6,777, among bacterial sequencing libraries, while NFZ4 had the lowest number of reads, i.e., 11,224, among fungal sequencing libraries. In the α-diversity analyses, the sequences per sample were rarefied to the same number (bacteria 6,777, fungi 11,224), which were randomly selected. Chao and Ace ((http://www.mothur.org) were calculated to characterize the community richness; Shannon index and Simpson index were calculated to characterize the community diversity. Rarefaction curves, reflecting the sequencing depth, were calculated using custom R scripts. To characterize the richness in a specific rhizosphere community, the custom R scripts were used to obtain Shannon-Wiener curve, Venn diagrams, and the microbial community bar plots. In the β-diversity analyses, R package vegan was used to obtain the heat map. Principal coordinate analyses (PCoA) utilizing the weighted and unweighted UniFrac distances were calculated using the pcoa() function of the R package Ape^45^.

The rhizosphere microbial communities of different regions/species were further compared using Anosim, Adonis, Metastats^46^, and LDA EffectSize (LEfSe)^12^, etc. Adonis is a function for the analysis and partitioning sums of squares using semimetric and metric distance matrices. Anosim is a distribution-free method of multivariate data analysis to test whether inter-group difference is significantly greater than intra-group one. The software vegan of R package was used to perform Adonis and Anosim. Metastats (http://metastats.cbcb.umd.edu) is a statistical method for detecting differentially abundant features between microbial communities, i.e., features that are enriched or depleted in one population versus another. LEfSe analysis was performed on http://huttenhower.sph.harvard.edu/galaxy. The differential features were identified on the OTU level. The three rhizosphere groups were used as the class of subjects.

## Additional Information

**How to cite this article**: Hao, D. C. *et al.* Unearthing microbial diversity of *Taxus* rhizosphere via MiSeq high-throughput amplicon sequencing and isolate characterization. *Sci. Rep.* **6**, 22006; doi: 10.1038/srep22006 (2016).

## Supplementary Material

Supplementary Information

Supplementary Table S1

Supplementary Table S2

Supplementary Table S3

Supplementary Table S4

Supplementary Table S5

Supplementary Table S6

## Figures and Tables

**Table 1:** Richness and diversity estimation of the 16S rRNA/ITS sequencing libraries from the MiSeq sequencing analysis.

Sample	OTUs	Ace	Chao	Shannon	Simpson	Coverage	Actual no. of reads
Bacteria(16SrRNA)							
NFXJ1-5	1082 ± 51.6 (901.6 ± 34.5*)	1407.6 ± 46.4 (1262.6 ± 53.5)	1410.4 ± 58.9 (1233.8 ± 65.6)	5.74 ± 0.03 (5.70 ± 0.04)	0.0081 ± 0.0008 (0.0081 ± 0.0008)	0.9711 ± 0.0050 (0.9519 ± 0.0025)	11843.8 ± 1740.0 (6777)
MDXJ1-5	1235.2 ± 68.8 (1083.6 ± 16.9)	1534.4 ± 35.1(1470 ± 12.7)	1506 ± 39.6 (1418.4 ± 27.4)	6.10 ± 0.01 (6.08 ± 0.02)	0.0046 ± 0.0001 (0.0046 ± 0.0001)	0.9659 ± 0.0083 (0.9444 ± 0.0004)	10626.8 ± 2178.5 (6777)
ZSXJ1-5	1200.6 ± 133.2 (1126 ± 30.4)	1586.2 ± 84.6 (1559.2 ± 53.8)	1552.6 ± 94.4 (1546 ± 77.8)	6.07 ± 0.09 (6.05 ± 0.05)	0.0056 ± 0.0003 (0.0056 ± 0.0003)	0.9498 ± 0.0131 (0.9391 ± 0.0030)	8378 ± 2451.8 (6777)
Fungi (ITS)							
NFZ1-5	650.6 ± 89.1 (558.8 ± 62.3)	833.6 ± 62.9 (792.4 ± 75.9)	798 ± 69.6 (763.8 ± 74.5)	3.73 ± 0.32 (3.71 ± 0.33)	0.0936 ± 0.0383 (0.0944 ± 0.0397)	0.989 ± 0.0041 (0.9819 ± 0.0019)	18387.2 ± 4886.4 (11224)
MDZ1-5	735.4 ± 85.5 (572.4 ± 57.3)	998.6 ± 64.6 (923.4 ± 78.8)	929 ± 74.6 (827.8 ± 98.4)	4.57 ± 0.21 (4.56 ± 0.21)	0.0258 ± 0.0063 (0.0256 ± 0.0062)	0.9906 ± 0.0015 (0.9812 ± 0.0017)	23886.4 ± 3531.9 (11224)
ZSZ1-5	960.2 ± 30.6 (760.4 ± 38.0)	1166.6 ± 42.0 (1116 ± 113.8)	1140.2 ± 40.6 (1056.6 ± 40.6)	4.85 ± 0.18 (4.83 ± 0.17)	0.0235 ± 0.0078 (0.0236 ± 0.0076)	0.9899 ± 0.0015 (0.9755 ± 0.0016)	24520.8 ± 2297.7 (11224)

The cutoff value is 0.03 (sequence identity 0.97). Chao and Ace are used to evaluate the community richness, while Shannon and Simpson are used to assess the community diversity. The values of mean ± SD of five samples are shown in the table. *values after rarefaction in the parentheses. NF, *T. mairei*; MD, *T. *×* media*; ZS, *T. cuspidata var. nana*. XJ, bacteria; Z, fungi.
