# Supplementary material for: Unearthing microbial diversity of Taxus rhizosphere via MiSeq high-throughput amplicon sequencing and isolate characterization
Source: Sci Rep. 2016 Apr 15;6:22006. doi: 10.1038/srep22006 (PMC4832182; doi:10.1038/srep22006)
Supplement: Supplementary Information [file srep22006-s1.pdf]

Unearthing microbial diversity of *Taxus* rhizosphere via MiSeq high-throughput amplicon sequencing and isolate characterization

Da Cheng Hao[1*], Si Meng Song[1], Wen Li Hu[1], Jun Mu[2*], Pei Gen Xiao[3]

[1]Biotechnology Institute, School of Environment and Chemical Engineering, Dalian Jiaotong University, Dalian 116028, China;

[2]College of Marine Science and Technology, Zhejiang Ocean University, Zhoushan 316022, China;

[3]Institute of Medicinal Plant Development, Chinese Academy of Medical Sciences, Beijing 100193, China;

*Correspondence: DCH, hao@djtu.edu.cn; JM, 2240254374@qq.com

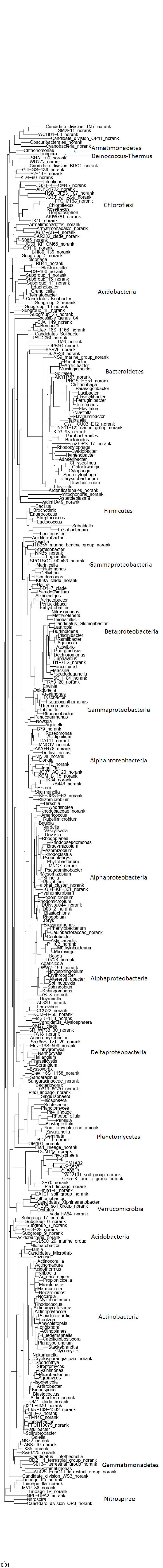

Armatimonadetes
Deinococcus-Thermus
Chloroflexi
Acidobacteria
Bacteroidetes
Firmicutes
Gammaproteobacteria
Betaproteobacteria
Gammaproteobacteria
Alphaproteobacteria
Alphaproteobacteria
Alphaproteobacteria
Deltaproteobacteria
Planctomycetes
Verrucomicrobia
Acidobacteria
Actinobacteria
Gemmatimonadetes
Nitrospirae
0.01

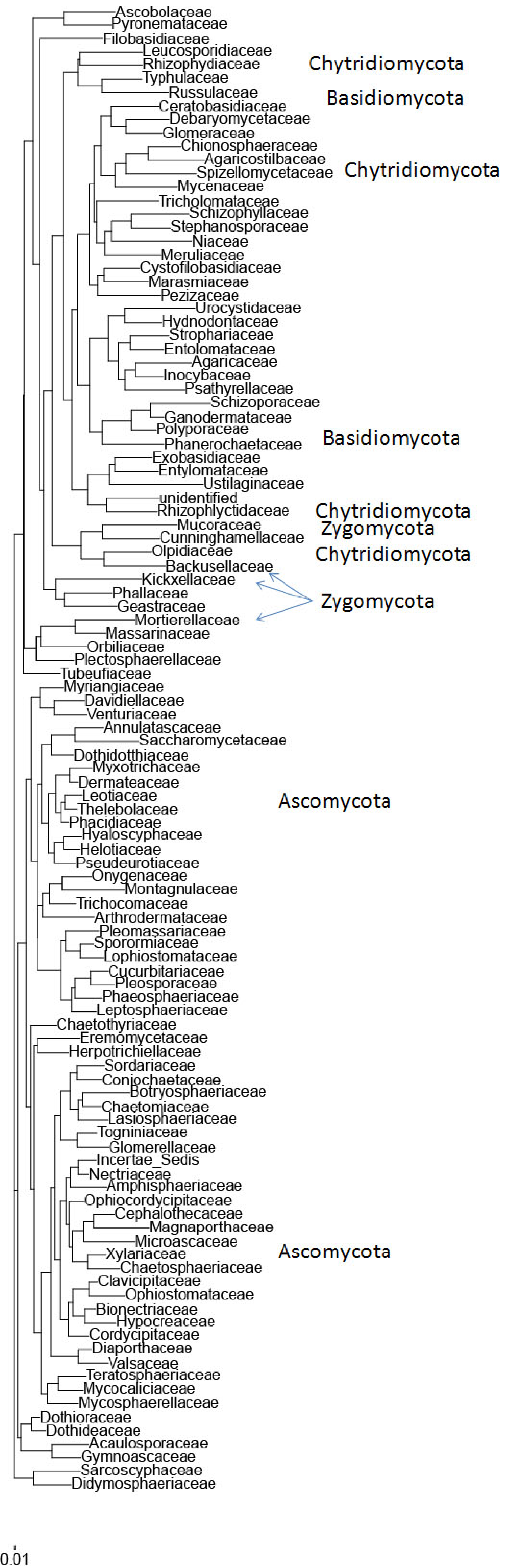
Ascobolaceae
Pyronemataceae
Filobasidiaceae
Leucosporidiaceae
Rhizophydiaceae
Typhulaceae
Russulaceae
Ceratobasidiaceae
Debaryomycetaceae
Glomeraceae
Chionosphaeraceae
Agaricostilbaceae
Spizellomycetaceae
Mycenaceae
Tricholomataceae
Schizophyllaceae
Stephanosporaceae
Niaceae
Meruliaceae
Cystofilobasidiaceae
Marasmiaceae
Pezizaceae
Urocystidaceae
Hydnodontaceae
Strophariaceae
Entolomataceae
Agaricaceae
Inocybaceae
Psathyrellaceae
Schizoporaceae
Ganodermataceae
Polyporaceae
Phanerochaetaceae
Exobasidiaceae
Entylomataceae
Ustilaginaceae
unidentified
Rhizophlyctidaceae
Mucoraceae
Cunninghamellaceae
Olpidiaceae
Backusellaceae
Kickxellaceae
Phallaceae
Geastraceae
Mortierellaceae
Massarinaceae
Orbiliaceae
Plectosphaerellaceae
Tubeufiaceae
Myriangiaceae
Davidiellaceae
Venturiaceae
Annulatascaceae
Saccharomycetaceae
Dothidotthiaceae
Myxotrichaceae
Dermateaceae
Leotiaceae
Thelebolaceae
Phacidiaceae
Hyaloscyphaceae
Helotiaceae
Pseudeurotiaceae
Onygenaceae
Montagnulaceae
Trichocomaceae
Arthrodermataceae
Pleomassariaceae
Sporormiaceae
Lophiostomataceae
Cucurbitariaceae
Pleosporaceae
Phaeosphaeriaceae
Leptosphaeriaceae
Chaetothyriaceae
Eremomycetaceae
Herpotrichiellaceae
Sordariaceae
Coniochaetaceae
Botryosphaeriaceae
Chaetomiaceae
Lasiosphaeriaceae
Togniniaceae
Glomerellaceae
Incertae_Sedis
Nectriaceae
Amphisphaeriaceae
Ophiocordycipitaceae
Cephalothecaceae
Magnaporthaceae
Microascaceae
Xylariaceae
Chaetosphaeriaceae
Clavicipitaceae
Ophiostomataceae
Bionectriaceae
Hypocreaceae
Cordycipitaceae
Diaporthaceae
Valsaceae
Teratosphaeriaceae
Mycocaliciaceae
Mycosphaerellaceae
Dothioraceae
Dothideaceae
Acaulosporaceae
Gymnoascaceae
Sarcoscyphaceae
Didymosphaeriaceae
Chytridiomycota
Basidiomycota
Chytridiomycota
Basidiomycota
Chytridiomycota
Zygomycota
Chytridiomycota
Zygomycota
Ascomycota
Ascomycota
0.01

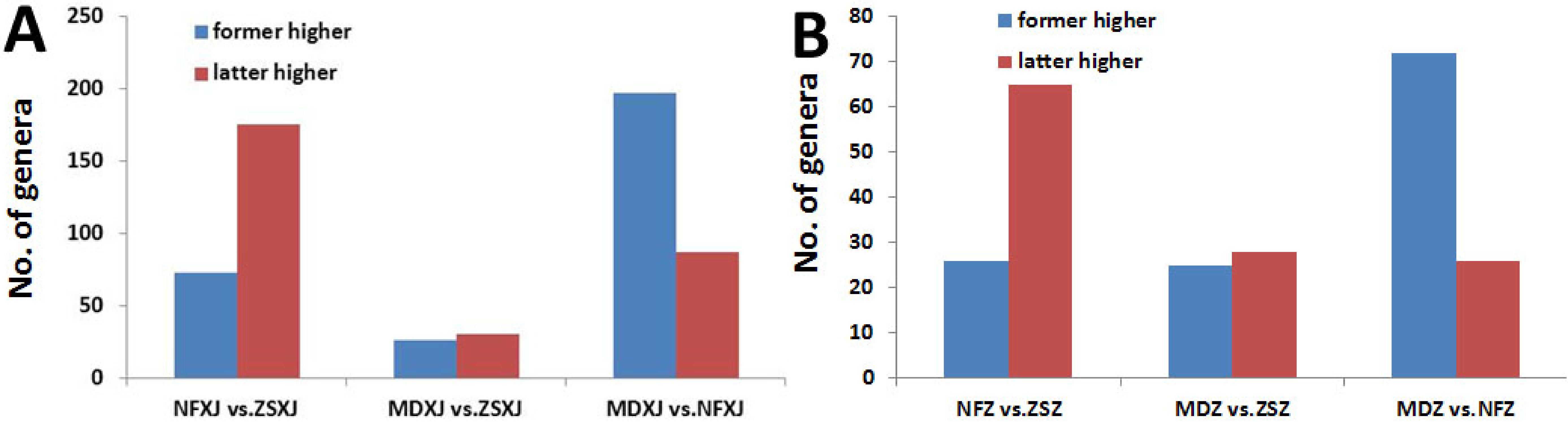
A
B
former higher
latter higher
No. of genera
250
200
150
100
50
0
NFXJ vs.ZSXJ
MDXJ vs.ZSXJ
MDXJ vs.NFXJ
80
70
60
50
40
30
20
10
0
NFZ vs.ZSZ
MDZ vs.ZSZ
MDZ vs.NFZ

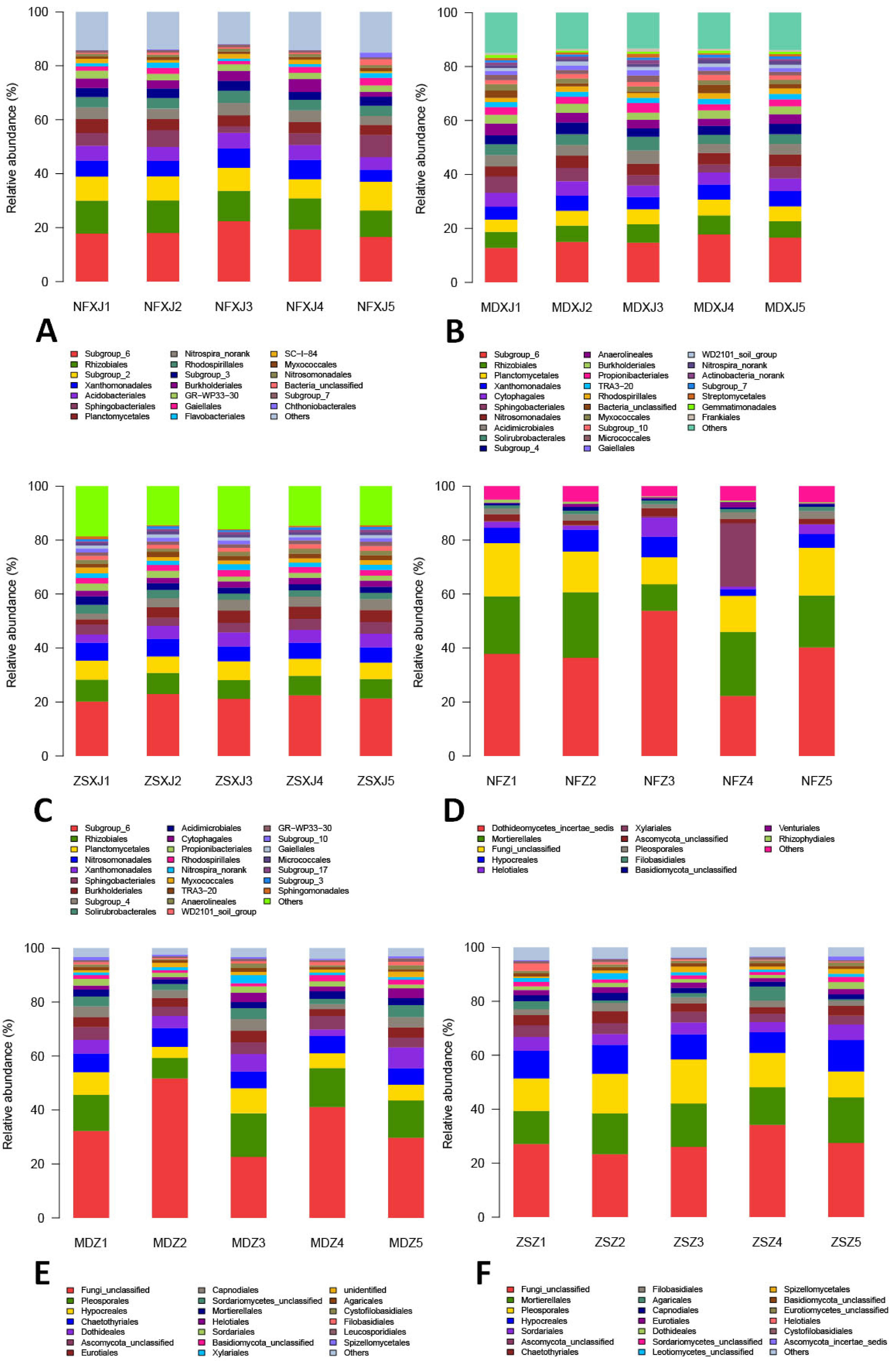
A
Relative abundance (%)
0
20
40
60
80
100
NFXJ1
NFXJ2
NFXJ3
NFXJ4
NFXJ5
Subgroup_6
Rhizobiales
Subgroup_2
Xanthomonadales
Acidobacteriales
Sphingobacteriales
Planctomycetales
Nitrospira_norank
Rhodospirillales
Subgroup_3
Burkholderiales
GR−WP33−30
Gaiellales
Flavobacteriales
SC−I−84
Myxococcales
Nitrosomonadales
Bacteria_unclassified
Subgroup_7
Chthoniobacterales
Others
B
MDXJ1
MDXJ2
MDXJ3
MDXJ4
MDXJ5
Subgroup_6
Rhizobiales
Planctomycetales
Xanthomonadales
Cytophagales
Sphingobacteriales
Nitrosomonadales
Acidimicrobiales
Solirubrobacterales
Subgroup_4
Anaerolineales
Burkholderiales
Propionibacteriales
TRA3−20
Rhodospirillales
Bacteria_unclassified
Myxococcales
Subgroup_10
Micrococcales
Gaiellales
WD2101_soil_group
Nitrospira_norank
Actinobacteria_norank
Subgroup_7
Streptomycetales
Gemmatimonadales
Frankiales
Others
C
ZSXJ1
ZSXJ2
ZSXJ3
ZSXJ4
ZSXJ5
Subgroup_6
Rhizobiales
Planctomycetales
Nitrosomonadales
Xanthomonadales
Sphingobacteriales
Burkholderiales
Subgroup_4
Solirubrobacterales
Acidimicrobiales
Cytophagales
Propionibacteriales
Rhodospirillales
Nitrospira_norank
Myxococcales
TRA3−20
Anaerolineales
WD2101_soil_group
GR−WP33−30
Subgroup_10
Gaiellales
Micrococcales
Subgroup_17
Subgroup_3
Sphingomonadales
Others
D
NFZ1
NFZ2
NFZ3
NFZ4
NFZ5
Dothideomycetes_incertae_sedis
Mortierellales
Fungi_unclassified
Hypocreales
Helotiales
Xylariales
Ascomycota_unclassified
Pleosporales
Filobasidiales
Basidiomycota_unclassified
Venturiales
Rhizophydiales
Others
E
MDZ1
MDZ2
MDZ3
MDZ4
MDZ5
Fungi_unclassified
Pleosporales
Hypocreales
Chaetothyriales
Dothideales
Ascomycota_unclassified
Eurotiales
Capnodiales
Sordariomycetes_unclassified
Mortierellales
Helotiales
Sordariales
Basidiomycota_unclassified
Xylariales
unidentified
Agaricales
Cystofilobasidiales
Filobasidiales
Leucosporidiales
Spizellomycetales
Others
F
ZSZ1
ZSZ2
ZSZ3
ZSZ4
ZSZ5
Fungi_unclassified
Mortierellales
Pleosporales
Hypocreales
Sordariales
Ascomycota_unclassified
Chaetothyriales
Filobasidiales
Agaricales
Capnodiales
Eurotiales
Dothideales
Sordariomycetes_unclassified
Leotiomycetes_unclassified
Spizellomycetales
Basidiomycota_unclassified
Eurotiomycetes_unclassified
Helotiales
Cystofilobasidiales
Ascomycota_incertae_sedis
Others

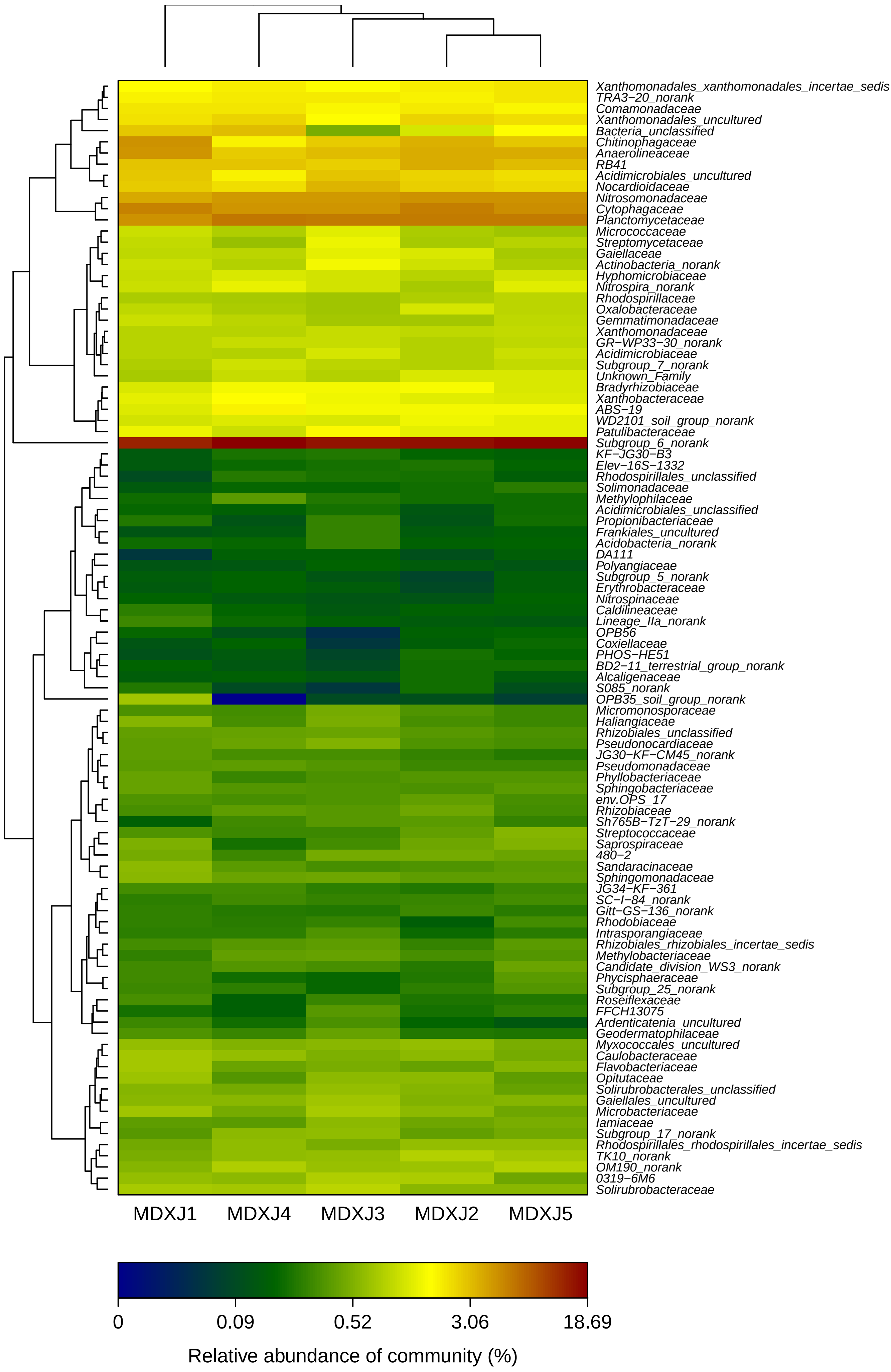

Xanthomonadales_xanthomonadales_incertae_sedis
TRA3−20_norank
Comamonadaceae
Xanthomonadales_uncultured
Bacteria_unclassified
Chitinophagaceae
Anaerolineaceae
RB41
Acidimicrobiales_uncultured
Nocardioidaceae
Nitrosomonadaceae
Cytophagaceae
Planctomycetaceae
Micrococcaceae
Streptomycetaceae
Gaiellaceae
Actinobacteria_norank
Hyphomicrobiaceae
Nitrospira_norank
Rhodospirillaceae
Oxalobacteraceae
Gemmatimonadaceae
Xanthomonadaceae
GR−WP33−30_norank
Acidimicrobiaceae
Subgroup_7_norank
Unknown_Family
Bradyrhizobiaceae
Xanthobacteraceae
ABS−19
WD2101_soil_group_norank
Patulibacteraceae
Subgroup_6_norank
KF−JG30−B3
Elev−16S−1332
Rhodospirillales_unclassified
Solimonadaceae
Methylophilaceae
Acidimicrobiales_unclassified
Propionibacteriaceae
Frankiales_uncultured
Acidobacteria_norank
DA111
Polyangiaceae
Subgroup_5_norank
Erythrobacteraceae
Nitrospinaceae
Caldilineaceae
Lineage_IIa_norank
OPB56
Coxiellaceae
PHOS−HE51
BD2−11_terrestrial_group_norank
Alcaligenaceae
S085_norank
OPB35_soil_group_norank
Micromonosporaceae
Haliangiaceae
Rhizobiales_unclassified
Pseudonocardiaceae
JG30−KF−CM45_norank
Pseudomonadaceae
Phyllobacteriaceae
Sphingobacteriaceae
env.OPS_17
Rhizobiaceae
Sh765B−TzT−29_norank
Streptococcaceae
Saprospiraceae
480−2
Sandaracinaceae
Sphingomonadaceae
JG34−KF−361
SC−I−84_norank
Gitt−GS−136_norank
Rhodobiaceae
Intrasporangiaceae
Rhizobiales_rhizobiales_incertae_sedis
Methylobacteriaceae
Candidate_division_WS3_norank
Phycisphaeraceae
Subgroup_25_norank
Roseiflexaceae
FFCH13075
Ardenticatenia_uncultured
Geodermatophilaceae
Myxococcales_uncultured
Caulobacteraceae
Flavobacteriaceae
Opitutaceae
Solirubrobacterales_unclassified
Gaiellales_uncultured
Microbacteriaceae
Iamiaceae
Subgroup_17_norank
Rhodospirillales_rhodospirillales_incertae_sedis
TK10_norank
OM190_norank
0319−6M6
Solirubrobacteraceae
MDXJ1
MDXJ4
MDXJ3
MDXJ2
MDXJ5
0
0.09
0.52
3.06
18.69
Relative abundance of community (%)

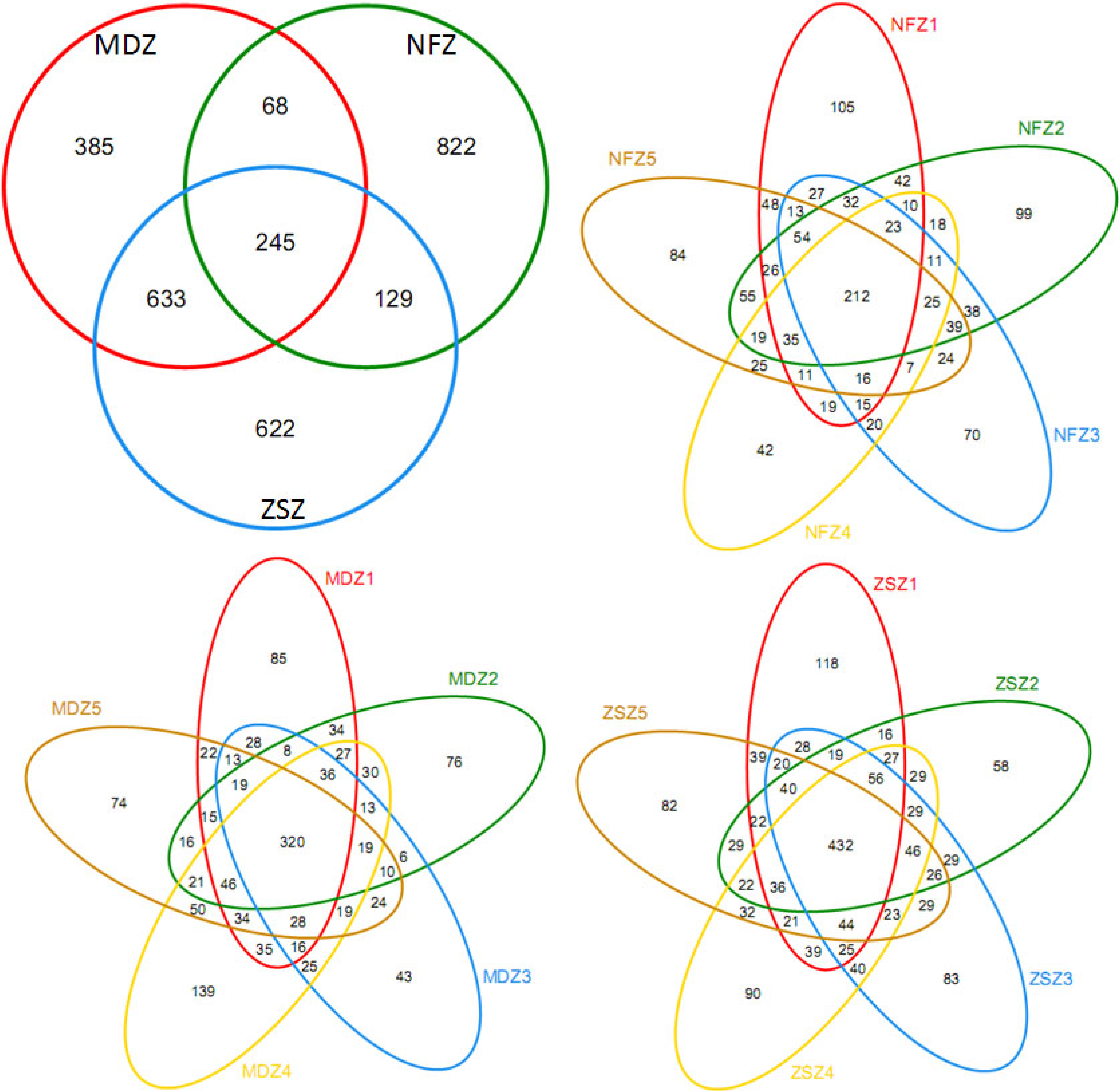
MDZ
NFZ
ZSZ
385
68
822
245
633
129
622
NFZ1
NFZ2
NFZ3
NFZ4
NFZ5
105
99
70
42
84
212
MDZ1
MDZ2
MDZ3
MDZ4
MDZ5
85
76
43
139
74
320
ZSZ1
ZSZ2
ZSZ3
ZSZ4
ZSZ5
118
58
83
90
82
432

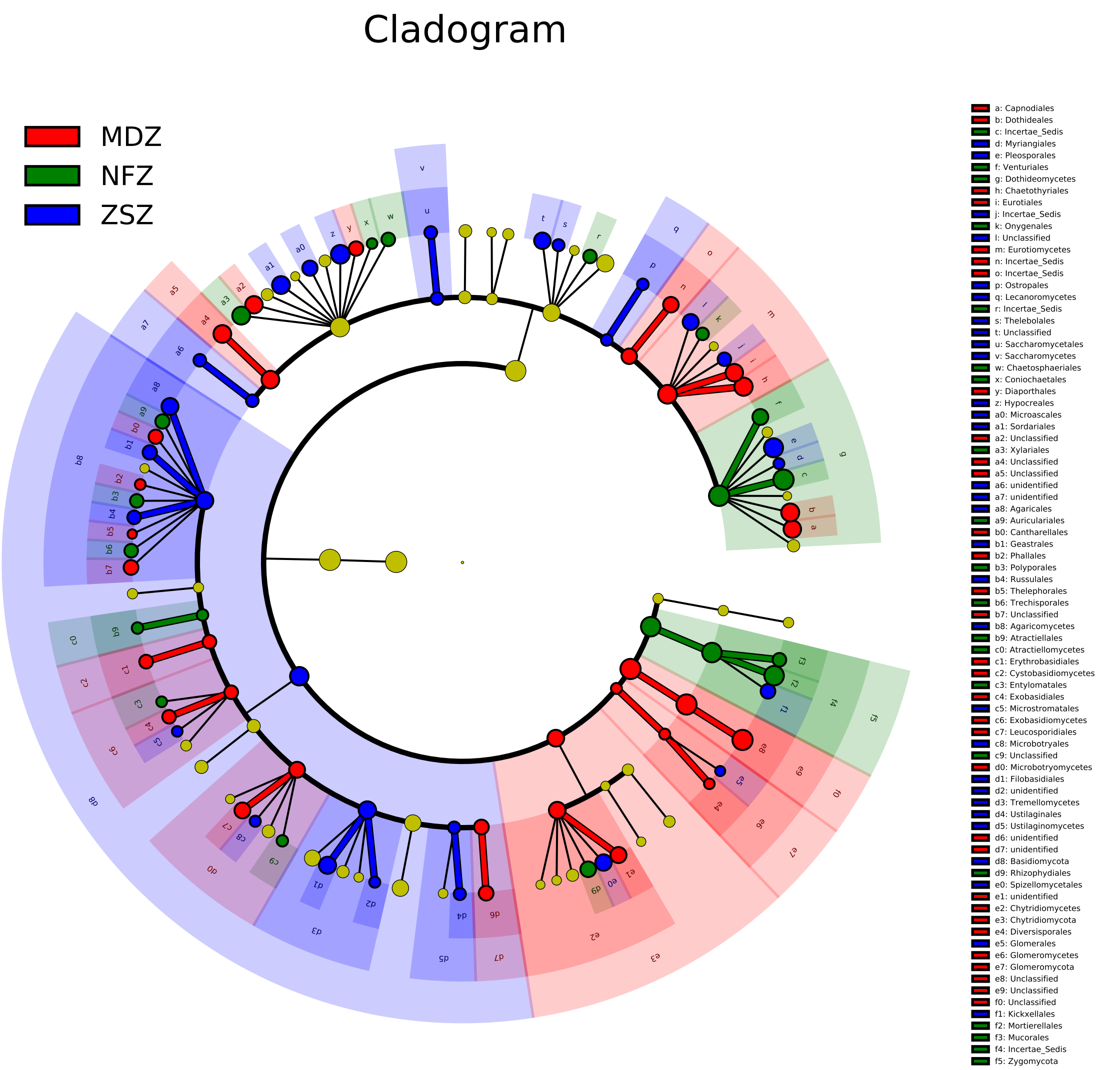
Cladogram
MDZ
NFZ
ZSZ
a: Capnodiales
b: Dothideales
c: Incertae_Sedis
d: Myriangiales
e: Pleosporales
f: Venturiales
g: Dothideomycetes
h: Chaetothyriales
i: Eurotiales
j: Incertae_Sedis
k: Onygenales
l: Unclassified
m: Eurotiomycetes
n: Incertae_Sedis
o: Incertae_Sedis
p: Ostropales
q: Lecanoromycetes
r: Incertae_Sedis
s: Thelebolales
t: Unclassified
u: Saccharomycetales
v: Saccharomycetes
w: Chaetosphaeriales
x: Coniochaetales
y: Diaporthales
z: Hypocreales
a0: Microascales
a1: Sordariales
a2: Unclassified
a3: Xylariales
a4: Unclassified
a5: Unclassified
a6: unidentified
a7: unidentified
a8: Agaricales
a9: Auriculariales
b0: Cantharellales
b1: Geastrales
b2: Phallales
b3: Polyporales
b4: Russulales
b5: Thelephorales
b6: Trechisporales
b7: Unclassified
b8: Agaricomycetes
b9: Atractiellales
c0: Atractiellomycetes
c1: Erythrobasidiales
c2: Cystobasidiomycetes
c3: Entylomatales
c4: Exobasidiales
c5: Microstromatales
c6: Exobasidiomycetes
c7: Leucosporidiales
c8: Microbotryales
c9: Unclassified
d0: Microbotryomycetes
d1: Filobasidiales
d2: unidentified
d3: Tremellomycetes
d4: Ustilaginales
d5: Ustilaginomycetes
d6: unidentified
d7: unidentified
d8: Basidiomycota
d9: Rhizophydiales
e0: Spizellomycetales
e1: unidentified
e2: Chytridiomycetes
e3: Chytridiomycota
e4: Diversisporales
e5: Glomerales
e6: Glomeromycetes
e7: Glomeromycota
e8: Unclassified
e9: Unclassified
f0: Unclassified
f1: Kickxellales
f2: Mortierellales
f3: Mucorales
f4: Incertae_Sedis
f5: Zygomycota

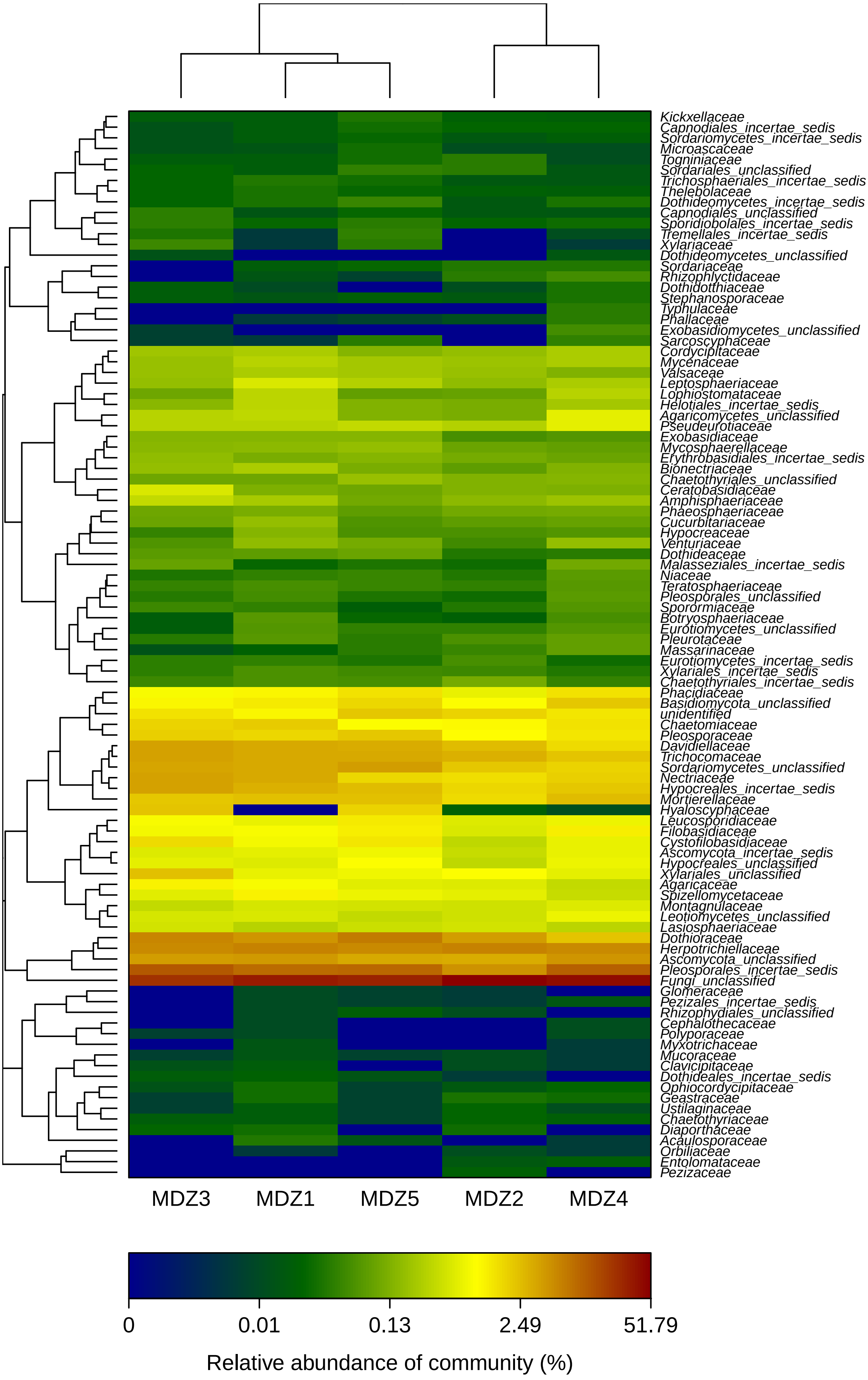
Kickxellaceae
Capnodiales_incertae_sedis
Sordariomycetes_incertae_sedis
Microascaceae
Togniniaceae
Sordariales_unclassified
Trichosphaeriales_incertae_sedis
Thelebolaceae
Dothideomycetes_incertae_sedis
Capnodiales_unclassified
Sporidiobolales_incertae_sedis
Tremellales_incertae_sedis
Xylariaceae
Dothideomycetes_unclassified
Sordariaceae
Rhizophlyctidaceae
Dothidotthiaceae
Stephanosporaceae
Typhulaceae
Phallaceae
Exobasidiomycetes_unclassified
Sarcoscyphaceae
Cordycipitaceae
Mycenaceae
Valsaceae
Leptosphaeriaceae
Lophiostomataceae
Helotiales_incertae_sedis
Agaricomycetes_unclassified
Pseudeurotiaceae
Exobasidiaceae
Mycosphaerellaceae
Erythrobasidiales_incertae_sedis
Bionectriaceae
Chaetothyriales_unclassified
Ceratobasidiaceae
Amphisphaeriaceae
Phaeosphaeriaceae
Cucurbitariaceae
Hypocreaceae
Venturiaceae
Dothideaceae
Malasseziales_incertae_sedis
Niaceae
Teratosphaeriaceae
Pleosporales_unclassified
Sporormiaceae
Botryosphaeriaceae
Eurotiomycetes_unclassified
Pleurotaceae
Massarinaceae
Eurotiomycetes_incertae_sedis
Xylariales_incertae_sedis
Chaetothyriales_incertae_sedis
Phacidiaceae
Basidiomycota_unclassified
unidentified
Chaetomiaceae
Pleosporaceae
Davidiellaceae
Trichocomaceae
Sordariomycetes_unclassified
Nectriaceae
Hypocreales_incertae_sedis
Mortierellaceae
Hyaloscyphaceae
Leucosporidiaceae
Filobasidiaceae
Cystofilobasidiaceae
Ascomycota_incertae_sedis
Hypocreales_unclassified
Xylariales_unclassified
Agaricaceae
Spizellomycetaceae
Montagnulaceae
Leotiomycetes_unclassified
Lasiosphaeriaceae
Dothioraceae
Herpotrichiellaceae
Ascomycota_unclassified
Pleosporales_incertae_sedis
Fungi_unclassified
Glomeraceae
Pezizales_incertae_sedis
Rhizophydiales_unclassified
Cephalothecaceae
Polyporaceae
Myxotrichaceae
Mucoraceae
Clavicipitaceae
Dothideales_incertae_sedis
Ophiocordycipitaceae
Geastraceae
Ustilaginaceae
Chaetothyriaceae
Diaporthaceae
Acaulosporaceae
Orbiliaceae
Entolomataceae
Pezizaceae
MDZ3
MDZ1
MDZ5
MDZ2
MDZ4
0
0.01
0.13
2.49
51.79
Relative abundance of community (%)

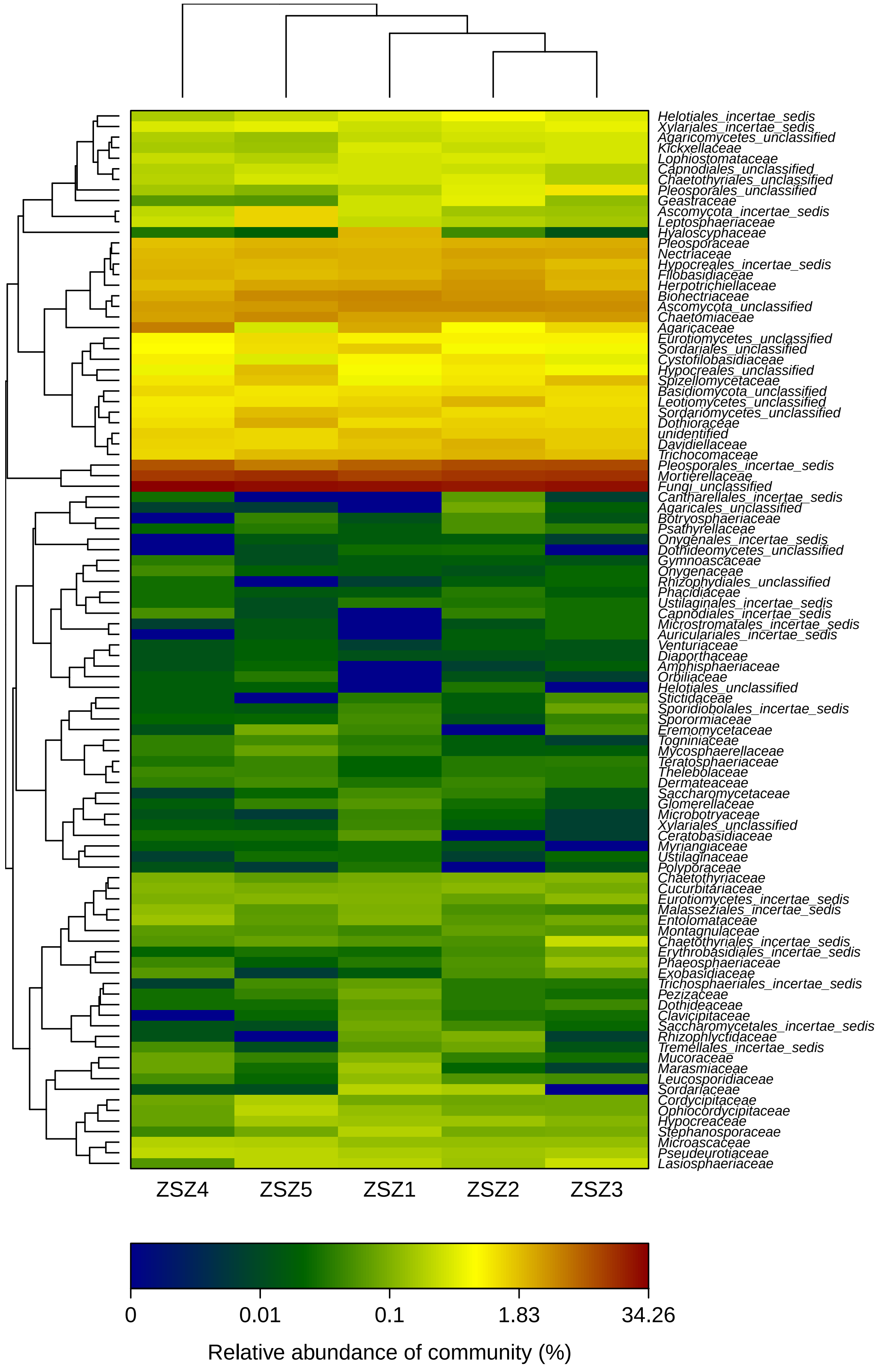

Helotiales_incertae_sedis
Xylariales_incertae_sedis
Agaricomycetes_unclassified
Kickxellaceae
Lophiostomataceae
Capnodiales_unclassified
Chaetothyriales_unclassified
Pleosporales_unclassified
Geastraceae
Ascomycota_incertae_sedis
Leptosphaeriaceae
Hyaloscyphaceae
Pleosporaceae
Nectriaceae
Hypocreales_incertae_sedis
Filobasidiaceae
Herpotrichiellaceae
Bionectriaceae
Ascomycota_unclassified
Chaetomiaceae
Agaricaceae
Eurotiomycetes_unclassified
Sordariales_unclassified
Cystofilobasidiaceae
Hypocreales_unclassified
Spizellomycetaceae
Basidiomycota_unclassified
Leotiomycetes_unclassified
Sordariomycetes_unclassified
Dothioraceae
unidentified
Davidiellaceae
Trichocomaceae
Pleosporales_incertae_sedis
Mortierellaceae
Fungi_unclassified
Cantharellales_incertae_sedis
Agaricales_unclassified
Botryosphaeriaceae
Psathyrellaceae
Onygenales_incertae_sedis
Dothideomycetes_unclassified
Gymnoascaceae
Onygenaceae
Rhizophydiales_unclassified
Phacidiaceae
Ustilaginales_incertae_sedis
Capnodiales_incertae_sedis
Microstromatales_incertae_sedis
Auriculariales_incertae_sedis
Venturiaceae
Diaporthaceae
Amphisphaeriaceae
Orbiliaceae
Helotiales_unclassified
Stictidaceae
Sporidiobolales_incertae_sedis
Sporormiaceae
Eremomycetaceae
Togniniaceae
Mycosphaerellaceae
Teratosphaeriaceae
Thelebolaceae
Dermateaceae
Saccharomycetaceae
Glomerellaceae
Microbotryaceae
Xylariales_unclassified
Ceratobasidiaceae
Myriangiaceae
Ustilaginaceae
Polyporaceae
Chaetothyriaceae
Cucurbitariaceae
Eurotiomycetes_incertae_sedis
Malasseziales_incertae_sedis
Entolomataceae
Montagnulaceae
Chaetothyriales_incertae_sedis
Erythrobasidiales_incertae_sedis
Phaeosphaeriaceae
Exobasidiaceae
Trichosphaeriales_incertae_sedis
Pezizaceae
Dothideaceae
Clavicipitaceae
Saccharomycetales_incertae_sedis
Rhizophlyctidaceae
Tremellales_incertae_sedis
Mucoraceae
Marasmiaceae
Leucosporidiaceae
Sordariaceae
Cordycipitaceae
Ophiocordycipitaceae
Hypocreaceae
Stephanosporaceae
Microascaceae
Pseudeurotiaceae
Lasiosphaeriaceae
ZSZ4
ZSZ5
ZSZ1
ZSZ2
ZSZ3
0
0.01
0.1
1.83
34.26
Relative abundance of community (%)

Supplementary information

Fig. S1 Maximum likelihood phylogenies inferred by FastTree 2. A, Phylogenetic relationship of the bacterial genera that are identified in *Taxus* rhizosphere, based on 16S rRNA sequences. B, Phylogenetic relationship of the fungal families that are identified in *Taxus* rhizosphere, based on ITS sequences. The scale bar represents a sequence divergence of 1%.

Fig. S2 Metastats results at the genus level. Metastats is able to rapidly and accurately compare large datasets generated from complex microbial communities to identify features that distinguish them. The number of genera that exhibited significantly different abundance in NF, ZS, and MD is shown. A, bacteria (XJ); B, fungi (Z).

Fig. S3 Relative abundance of different microbial orders in *Taxus* rhizosphere. A-C, bacteria. Rhizobiales, Rhodospirillales, and Sphingomonadales belong to Alphaproteobacteria; Burkholderiales and Nitrosomonadales belongs to Betaproteobacteria; Xanthomonadales belongs to Gammaproteobacteria; Myxococcales belongs to Deltaproteobacteria; Sphingobacteriales belongs to Sphingobacteriia (Bacteroidetes); Flavobacteriales belongs to Flavobacteriia (Bacteroidetes); Cytophagales belongs to Cytophagia (Bacteroidetes); Gaiellales, Solirubrobacterales, Propionibacteriales, Micrococcales, Streptomycetales, Frankiales, and Acidimicrobiales belong to Actinobacteria; Anaerolineales belongs to Anaerolineae (Chloroflexi); Chthoniobacterales belongs to Spartobacteria (Verrucomicrobia). D-F, fungi. The order Mortierellales belongs to unclassified class (Zygomycota); Pleosporales, Dothideales, Venturiales, and Capnodiales belong to Dothideomycetes (Ascomycota); Hypocreales, Xylariales, and Sordariales belong to Sordariomycetes (Ascomycota); Chaetothyriales and Eurotiales belong to Eurotiomycetes (Ascomycota); Helotiales belongs to Leotiomycetes (Ascomycota); Filobasidiales and Cystofilobasidiales belong to Tremellomycetes (Basidiomycota); Agaricales belongs to Agaricomycetes (Basidiomycota); Leucosporidiales belongs to Microbotryomycetes (Basidiomycota); Spizellomycetales and Rhizophydiales belong to Chytridiomycetes (Chytridiomycota).

Fig. S4 Heat maps showing bacterial family frequency distribution in MD. The top 100 abundant families are shown. The different color intensities represent the relative bacterial abundance in each rhizosphere sample. The clustering along y axis is based on abundance of family reads present in each rhizosphere sample (x axis); the clustering along x axis is based on the similarity of the inter-sample abundance.

Fig. S5 Venn diagrams showing the common and exclusive fungal OTUs of the *Taxus* rhizospheres. MD, *T.* ×*media*; ZS, *T. cuspidata var. nana*; NF, *T. mairei*.

Fig. S6 LEfSe results on *Taxus* rhizosphere microbiomes. The cladogram reports the taxonomic representation of statistically and biologically consistent differences between MD, ZS, and NF fungal communities. Differences are represented in the color of the most abundant class (red indicating MD, green NF, blue ZS, yellow non-significant). Each circle's diameter is proportional to the taxon's abundance.

Fig. S7 Heat maps showing fungal family frequency distribution in the MD rhizospheres. The top 100 abundant families are shown. The different color intensities represent the relative bacterial abundance in each rhizosphere sample. The clustering along y axis is based on abundance of family reads present in each rhizosphere sample (x axis); the clustering along x axis is based on the similarity of the inter-sample abundance. Vegan of R package was used for distance calculation and clustering analysis.

Fig. S8 Heat maps showing fungal family frequency distribution in ZS. The top 100 abundant families are shown. The different color intensities represent the relative bacterial abundance in each rhizosphere sample. The clustering along y axis is based on abundance of family reads present in each rhizosphere; the clustering along x axis is based on the similarity of the inter-sample abundance.

Table S1 Taxonomy and abundance of all 16S rRNA-based OTUs

Table S2 Taxonomy and abundance of all ITS-based OTUs

Table S3 Bacterial clades that consistently explain the statistically significant differences between NF, ZS, and MD

Table S4 Fungal clades that consistently explain the statistically significant differences between NF, ZS, and MD

Table S5 Relative abundance of fungi genera that are known to have paclitaxel-producing species

Table S6 Examples of rare fungi genera that are shared by MD and ZS but are not found in NF rhizosphere
